# Targeting immunosenescence for improved tumor immunotherapy

**DOI:** 10.1002/mco2.777

**Published:** 2024-10-28

**Authors:** Zaoqu Liu, Lulu Zuo, Zhaokai Zhou, Shutong Liu, Yuhao Ba, Anning Zuo, Yuqing Ren, Chuhan Zhang, Yukang Chen, Hongxuan Ma, Yudi Xu, Peng Luo, Quan Cheng, Hui Xu, Yuyuan Zhang, Siyuan Weng, Xinwei Han

**Affiliations:** ^1^ Department of Interventional Radiology The First Affiliated Hospital of Zhengzhou University Zhengzhou China; ^2^ Interventional Institute of Zhengzhou University Zhengzhou China; ^3^ Interventional Treatment and Clinical Research Center of Henan Province Zhengzhou China; ^4^ Institute of Basic Medical Sciences Chinese Academy of Medical Sciences and Peking Union Medical College Beijing China; ^5^ Center for Reproductive Medicine The First Affiliated Hospital of Zhengzhou University Zhengzhou China; ^6^ Department of Urology The First Affiliated Hospital of Zhengzhou University Zhengzhou China; ^7^ Department of Respiratory and Critical Care Medicine The First Affiliated Hospital of Zhengzhou University Zhengzhou China; ^8^ Department of Oncology The First Affiliated Hospital of Zhengzhou University Zhengzhou China; ^9^ Department of Kidney Transportation The First Affiliated Hospital of Zhengzhou University Zhengzhou China; ^10^ Department of Neurology The First Affiliated Hospital of Zhengzhou University Zhengzhou China; ^11^ Department of Oncology Zhujiang Hospital Southern Medical University Guangzhou China; ^12^ Department of Neurosurgery Xiangya Hospital Central South University Changsha China

**Keywords:** immune responses, immunosenescence, immunotherapy, inflammaging, older patients, therapeutic strategies, tumor immune microenvironment

## Abstract

Tumor immunotherapy has significantly transformed the field of oncology over the past decade. An optimal tumor immunotherapy would ideally elicit robust innate and adaptive immune responses within tumor immune microenvironment (TIME). Unfortunately, immune system experiences functional decline with chronological age, a process termed “immunosenescence,” which contributes to impaired immune responses against pathogens, suboptimal vaccination outcomes, and heightened vulnerability to various diseases, including cancer. In this context, we will elucidate hallmarks and molecular mechanisms underlying immunosenescence, detailing alterations in immunosenescence at molecular, cellular, organ, and disease levels. The role of immunosenescence in tumorigenesis and senescence‐related extracellular matrix (ECM) has also been addressed. Recognizing that immunosenescence is a dynamic process influenced by various factors, we will evaluate treatment strategies targeting hallmarks and molecular mechanisms, as well as methods for immune cell, organ restoration, and present emerging approaches in immunosenescence for tumor immunotherapy. The overarching goal of immunosenescence research is to prevent tumor development, recurrence, and metastasis, ultimately improving patient prognosis. Our review aims to reveal latest advancements and prospective directions in the field of immunosenescence research, offering a theoretical basis for development of practical anti‐immunosenescence and anti‐tumor strategies.

## Graphical abstract

Immune system of human organism has a well‐defined structural hierarchy. Over time, human organism undergoes substantial transformation through influence of immunosenescence. Advanced insight into immunosenescence and immunotherapy has facilitated a paradigm shift in treatment of elderly oncology patients. Anti‐immunosenescence and anti‐tumor strategies are closely interrelated and jointly improve therapeutic patterns and quality of life in oncology for older patients.

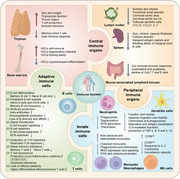

## 1 INTRODUCTION

With the progressive increase in global aging population, innovative tactics must be adopted to ensure sustained fitness and well‐being. It is unequivocal that immune system exhibits differential behavior in older individuals compared to younger ones. The prevailing belief is that this disparity not only increases susceptibility to infectious diseases but also results in prevalence of various degenerative conditions, particularly neurodegeneration, cancer, cardiovascular diseases, and autoimmune disorders.^1^, ^2^ Given that median age of cancer diagnosis is 66 years, preventing and eliminating tumors in older adults is evolving into a far‐reaching public health concern.

Therapeutic modalities that harness immune system to identify and eradicate cancer, collectively referred to as cancer immunotherapy, have revolutionized cancer treatment and revitalized the field of tumor immunology. It implies that comprehending immune infiltrates within tumor immune microenvironment (TIME) is crucial for enhancing response rates and devising novel therapeutic strategies for cancer treatment through immunotherapy. Various immunotherapies, such as adoptive cell transfer (ACT) and immune checkpoint inhibitors (ICIs), have achieved durable clinical responses.^3^ However, immunosenescence as a highly dynamic, multifactorial, and sophisticated process involving numerous regulatory functions at diverse levels recently captured considerable attention in tumor research.^4^ In young and healthy individuals, neutrophils, monocytes, or macrophages, antigen‐presenting cells (APCs), natural killer (NK) cells, B cells, and T cells work together to eliminate impaired cells and prevent malignant tissue proliferation. In contrast, a stepwise decrease in integral immune functions is characteristic of immunosenescence, which directly facilitates senescence‐associated secretory phenotype (SASP), inflammaging, and eventually, tumors.^5^, ^6^ Within this framework, hallmarks and molecular mechanisms of immunosenescence will be explained to monitor its course and predict switch to pathological states. By tracking immunosenescence at various levels, potential fresh targets to address immunosenescent disorders may be revealed.^7^

**FIGURE 1:**
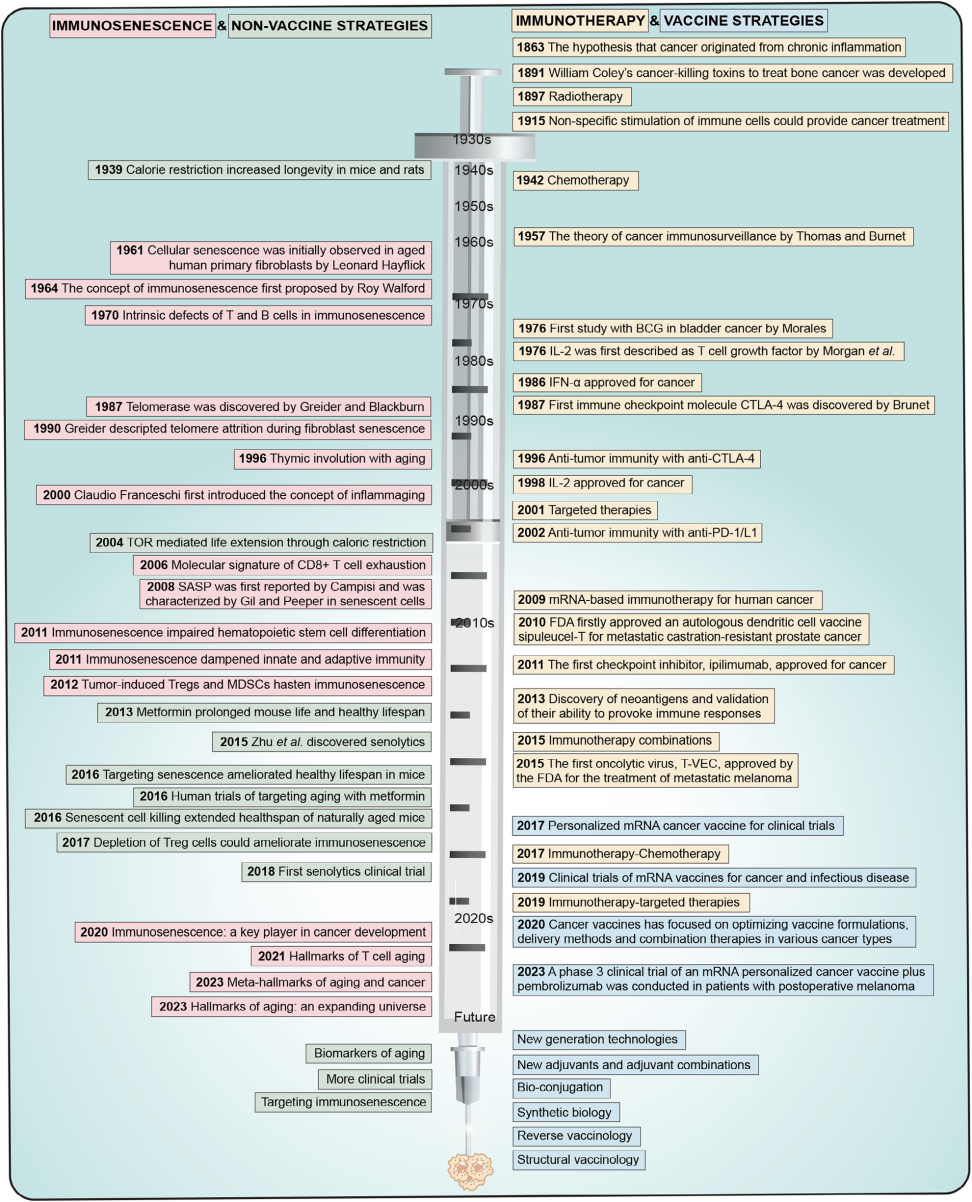
A timeline of events related to immunosenescence and immunotherapy. In this figure, we summarize the key discoveries regarding immunosenescence and immunotherapy. The increased understanding of immunosenescence and immunotherapy has facilitated a shift in the treatment paradigm for elderly tumor patients. Vaccine and non‐vaccine strategies to combat immunosenescence await further exploration and may offer outstanding opportunities for cancer patients.

In this review, we commence with a comprehensive deconstruction of immunosenescence, examining 12 hallmarks and five molecular mechanisms across various organizational levels, ranging from molecular to meta‐organismal. This analysis elucidates alterations in immune system at a molecular level. Building on this foundation, we propose immunosenescence‐associated changes in immune cells and organs, which unmask impaired immune responses in both innate and adaptive immunity, thereby highlighting manifestations of immunosenescence at cellular and organ levels. Furthermore, role of immunosenescence in tumorigenesis is examined in detail, elucidating its impact at disease level. Correspondingly, treatment strategies targeting aforementioned immunosenescent hallmarks, molecular mechanisms, cells, and organs are explored to halt cancer progression prior to tumor. We then delineate recent progresses in the field of immunosenescence as they pertain to tumor immunotherapy. These advancements encompass immunosuppression‐reducing treatments, immune checkpoint blockade, ACT, bi‐specific T‐cell engagers (BiTEs), and cancer vaccines, all of which hold promise for optimizing immune responses within a compromised environment. By embracing an integrated and comprehensive treatment strategy against tumors during immunosenescence, society could enhance quality of life for the elderly population, which is projected to increase significantly in both numbers and proportion in the forthcoming decades. Herein, this review paints a nuanced picture of immunosenescence, pointing us in direction of potential oncology immunotherapy (Figure 1).

## 2 UNTWINING OF IMMUNOSENESCENCE

### 2.1 Hallmarks of immunosenescence

Immunosenescence, the decay in immune system function and composition, is influenced by genetic, environmental, and metabolic elements.^8^ While its mechanisms are not fully agreed upon, the field recognizes common hallmarks of immunosenescence, which share three traits: (1) time‐dependent variations align with immunosenescence. (2) Potential to aggravate immunosenescence byexperimentally stirring these features. (3) Progress in countering or altering immunosenescencethrough targeted therapies.^9^ These hallmarks relate to discrepancies in dreadful clinical outcomes between the young and old.^10^ They are considered actionable targets for therapeutic interventions.

#### 2.1.1 Genomic instability and telomere attrition

*Genomic instability*: Cells frequently encounter DNA damage factors, comprising endogenous factors (e.g., DNA replication errors) and exogenous factors (e.g., irradiation, oxidative stress, and chemical mutagens). DNA‐related alterations including damage response and repair, mutations, replication stress, translocations, chromosomal aberrations, micronuclei, and fragment changes, could have effects on genes and transcriptional pathways, ultimately leading to defective biological functions. Additionally, DNA repair networks functionally decline with advancing age.^11^ DNA, as a vehicle for genetic information, accumulates damage over time, disrupting regular cell activity and tissue equilibrium, posing the consequence of immunosenescence. Large‐scale DNA transformations have been utilized to indicate a cell's biological state.^7^

*Telomere attrition*: Telomeres, nucleoprotein complexes situated at the ends of lineage chromosomes, are momentous for preventing DNA degradation, damage, and unwanted recombination in eukaryotes.^7^ They modulate telomere replication regulation, telomere capping, and higher‐order structural determination of telomeric chromatin. Derecapping of protective protein complexes from telomeres triggers DNA damage responses and undesirable DNA repair.^12^, ^13^ During DNA replication, the replication machinery is incapable of replicating telomeric DNA entirely, causing telomeres to shorten each time, namely the end‐replication problem.^14^ It could lead to DNA breakage and replicative senescence as telomeric DNA is shortened below a certain threshold.^15^ Therefore, telomere attrition could be utilized as a read‐out for assaying immunosenescence.^16^

#### 2.1.2 Epigenetic alterations and loss of proteostasis

*Epigenetic alterations*: Multicellular organisms’ cells are genetically homogeneous but heterogeneous in structure and function by virtue of gene expression differences are epigenetic since they are temporarily heritable without entailing DNA mutations.^17^ Epigenetic mechanisms critically manipulate proprietary biological processes by controlling gene transcription and translation, leading to increased transcriptional noise and abnormal mRNA production and maturation. Epigenetic dysregulation, including altered genomic DNA methylation (e.g., hypermethylated tumor suppressor genes), aberrant histone modifications (e.g., reduced H3K9 or H3K27 trimethylation), heterochromatin deletions, three‐dimensional genome structural reorganization, and deregulated RNA modifications (e.g., dysregulated non‐coding RNA), is broadly explored to be an immunosenescence hallmark.^18^

*Loss of proteostasis*: Immunosenescence expands the risk of aberrant protein aggregation due to progressive proteostasis loss.^19^ The proteostasis network consists of following core members: (1) molecular chaperones and co‐chaperones facilitate highly efficient folding and prevent misfolded proteins from aggregating. (2) Autophagy–lysosome and ubiquitin–proteasome systems, which are primary quality control pathways.^20^ (3) Protein misfolding mobilizes stress responses such as heat shock responses, endoplasmic reticulum unfolded protein response (UPR), and mitochondrial UPR.^21^ Immunosenescence, linked to decreased proteostasis network function, contributes to accumulated ubiquitinated, misfolded, and oxidized protein.^20^ It has been demonstrated that chaperone status, autophagy–lysosome system function, ubiquitin–proteasome system activity, as well as UPR in the endoplasmic reticulum and mitochondria, could be evolved into markers of immunosenescence.^7^

#### 2.1.3 Disabled autophagy and mitochondria dysfunction

*Disabled autophagy*: Autophagy refers to the process where cytoplasmic material is enclosed in a double‐membrane vesicle, the autophagosome, which merges with lysosomes for digestion.^22^ Accordingly, autophagy, related to protein homeostasis and impacting non‐proteinaceous macromolecules (e.g., glycogen), whole organelles, and pathogens, is a prominent factor in the decline of immune function during immunosenescence, affecting organelle turnover.^9^ Cassidy et al. have provided evidence that systemic autophagy inhibition induces premature manifestation of phenotypes and pathologies associated with immunosenescence in mammalian subjects.^23^ Moreover, autophagy could boost cell resistance to harsh conditions, for example, autophagy in fibroblasts may potentially augment tropic support to malignant cells and even subvert immuno‐surveillance. Collectively, disabled autophagy is justified as a characteristic feature of immunosenescence.^24^

*Mitochondria dysfunction*: Mitochondria, integral organelles within cellular structures, are involved in most cell activities containing cell signaling, energy supply, apoptosis regulation, calcium homeostasis, and multiple biosynthetic pathways. The mitochondrial genome, known as mitochondrial DNA (mtDNA), encodes an array of genes including proteins, ribosomal RNAs, and transfer RNAs. Furthermore, reactive oxygen species (ROS), largely launched through mitochondrial aerobic respiration, mainly produced in mitochondrial respiratory chain complexes I and III, could sponsor oxidative damage to mtDNA.^25^ The concomitant mtDNA mutations could trigger defective respiratory chain components that spark more ROS, which instigates a vicious spiral of ROS accumulation, mtDNA mutations, disequilibrium of mitochondrial functions, and acceleration of immunosenescence.^26^

#### 2.1.4 Deregulated nutrient‐sensing and cellular senescence

*Deregulated nutrient‐sensing*: The nutrient‐sensing network, such as the insulin/insulin growth factor‐1 (IGF‐1) signaling pathway, acts as a central accommodator of cellular energy and metabolic homeostasis, as well as diverse biological processes incorporating mRNA biogenesis, protein synthesis, mitochondrial biogenesis, proteasomal activity, and glucose, nucleotide, and lipid metabolism.^27^ The network primes metabolic or cellular defense pathways under stress and nutrient shortage circumstances. Nevertheless, it has been observed that a large number of nutrient sensors and targets undergo downregulation, a process correlated with immunosenescence, giving rise to a deregulated metabolism.^28^ Furthermore, disorders in nutrient uptake mechanisms could perturb homeostasis at cellular level. Recent studies have revealed that a genetic reduction in activity of nutrient‐sensing network components could potentially prolong a healthy lifespan in a spectrum of animal models.^29^

*Cellular senescence*: Cellular senescence is a response elicited from stress and injury such as DNA damage, replicative stress, oxidative stress, oncogene activation, organelle stress, etc.^30^ Cells will transition into a state of stable, permanent cell cycle arrest with flattened and enlarged macromolecular changes in culture.^31^, ^32^ Highly heterogeneous though, senescent cells share three key features: cell cycle arrest, resistance against apoptosis, and a hypersecretory, pro‐inflammatory phenotype, commonly referred to as SASP.^33^ Cellular senescence activation is saliently mediated through cell cycle proteins, cyclin‐dependent kinases (CDKs), CDK inhibitors, and retinoblastoma oncogenes (RBs), respectively.^30^, ^34^ The downstream signaling cascades evoked by immunosenescence triggers eventually converge on p53/p21^CIP^ and/or p16^INK4a^/pRB pathways, which dynamically interact with CDKs to suppress cell cycle, accordingly both p16^INK4a^ and p21^CIP1^ could be applied for detection of immunosenescence in vivo.^35^

#### 2.1.5 Stem cell exhaustion and altered intercellular communication

*Stem cell exhaustion*: The phenomenon of stem cell exhaustion, both quantitative and qualitative decreases in stem cell function throughout an organism's lifespan, is considered a momentous contributor to organismal immunosenescence.^36^ In the juvenile state, stem cells pertain to maintaining tissue homeostasis by facilitating tissue renewal and repair.^37^ The potential exists for induction of cellular de‐differentiation and plasticity by employing secreted cytokines, and growth factors to restructure microenvironment and extracellular matrix (ECM).^38^ Inspiringly, immunosenescent phenotypes could be reversed by stem cell rejuvenation in vivo, which could be a robust tool with the possibility to boost stem cell function and shift anti‐immunosenescence landscapes.^39^

*Altered intercellular communication*: Immunosenescence is connected with incremental changes in intercellular communication that hamper homeostasis and hormonal regulation. As a result, immunosenescence engages deficiencies in neural, neuroendocrine, and hormonal signaling pathways, containing adrenergic, dopaminergic, insulin/IGF‐1 system, renin–angiotensin system, among others. Importantly, modification in intercellular communication also encompasses short‐acting extracellular molecules, soluble factors, cell‐bound receptors, and cell‐to‐cell interactions during the process of immunosenescence.^40^ Dysregulation of intercellular communication links chronic inflammatory response weakened immunosurveillance against pathogens and premalignant cells, and altered bidirectional communication between human genome and microbiome to dysbiosis.^9^ Besides, the nexus between intercellular communication derangements and ECM disruption during immunosenescence is still in non‐stop exploration, intending to further elaborate communication systems between cells.

#### 2.1.6 Chronic inflammation and dysbiosis

*Chronic inflammation*: During immunosenescence, inflammation is chronically exacerbated, as evidenced by the elevated levels of circulating inflammatory cytokines and biomarkers. It is typified by elevated serum concentrations of inflammatory agents such as tumor necrosis factor (TNF), interleukin (IL)‐6, IL‐8, and other pro‐inflammatory cytokines.^41^ Chronic inflammation invokes intrinsic stimulus to most immune cells, thereby attenuating their immune responses.^42^ Additionally, chronic inflammation is common in immunosenescence‐related diseases and phenotypes, tissue senescence across several species, and is linked to increased mortality and multimorbidity risk.^43^ In head‐to‐head comparisons, lower peripheral blood levels of inflammatory cytokines are positively correlated with favorable fitness and diminished death risk in the elderly.^44^

*Dysbiosis*: It is acknowledged that composition of each individual's gut microbiota grows more unique ascribed to immune regulation, inflammation, and immunosenescence. Recently, gut microbes have appeared as a pivotal component in a myriad of physiological processes, including pathogen resistance, essential metabolite production, nutrient digestion and absorption, etc. Gut microbes also communicate with central and peripheral nervous systems, together with additional remote organs, facilitating comprehensive maintenance of host fitness.^45^ However, a breakdown in bidirectional communication between bacteria and the host could lead to dysbiosis and manifold immunosenescence‐related diseases, including cancer.^46^ Encouragingly, studies have verified that immunosenescent physiological transformations may have a far‐reaching influence on gut microbes of older individuals, independent of dietary and lifestyle variations.^9^

### 2.2 Molecular mechanisms underlying immunosenescence

#### 2.2.1 Antigen exposure and oxidative stress

*Antigen exposure*: Studies suggest that viruses, particularly human cytomegalovirus, could induce latent and chronic infections, thereby significantly influencing T‐cell compartments.^47^ Notably, human cytomegalovirus seropositivity is associated with an inversion of CD4/CD8 T‐cell ratio, primarily through chronic expansion of CD8CD28^+−^ effector T cells.^48^ These pro‐inflammatory cells, produce substantial amounts of interferon‐gamma (IFN‐γ) and TNF, but exhibit limited proliferative capacity upon antigenic stimulation and, consequently are classified as “senescent” cells.^49^ As specialized effector cells, they exhibit an increased susceptibility to apoptosis, which correlates with elevated mortality rates among the elderly population.^50^, ^51^, ^52^ Special attention has been paid to the fact that lifelong chronic antigen exposure exhausts T lymphocytes and diminishes T‐cell repertoire, resulting in inflammation and poor responses to new antigens. This phenomenon further drives immune system toward immunosenescence in elderly individuals.^53^

*Oxidative stress*: Many studies have demonstrated that oxidative damage increases with age across various tissues and species, and that adjusting respiration could extend lifespan in model organisms.^54^, ^55^, ^56^, ^57^ Research conducted by Pangrazzi et al. indicates that senescence modifies molecules in bone marrow that are required to maintain memory T cells and plasma cells. Elevated ROS levels in cells drive harmful senescence effects, including inflammaging as well as senescence‐related diseases.^58^, ^59^, ^60^ The accumulation of ROS results from impaired mitochondrial protein repair and degradation, which causes increased oxidative stress. This oxidative stress, coupled with reduced proteasome activity, exacerbates protein damage and functional decline, thereby giving rise to inflammaging and cellular senescence.^61^, ^62^

#### 2.2.2 Inflammaging and SASP

There is potential for any cell to senescence and secrete inflammatory signals that mark themselves for destruction due to mutation damage. However, with aging, excessive damage causes even immune cells to senesce, disrupting this wound‐healing process. Thus, not only do these immunosenescent cells inadequately perform necessary cleanup, but also exacerbate inflammation as well as further damage to adjacent healthy tissue.^63^ This phenomenon termed “inflammaging,” refers to elevated self‐reactivity in older adults, resulting in a classic chronic, low grade, and systemic sterile inflammatory state.^64^ Inflammaging is majorly triggered by stimuli such as stress, mechanical trauma, and ischemia, and occurs without pathogens, releasing danger‐associated molecular patterns. Consequently, they are detected by innate immune receptors such as Toll‐like receptors (TLRs) and nucleotide‐binding oligomerization domain (NOD)‐like receptor family, pyrin domain containing 3, activating signaling pathways that lead to inflammation.^65^

Senescent immune cells release a plethora of soluble factors including pro‐inflammatory cytokines, proteases, growth factors, angiogenic factors, chemokines, matrix metalloproteinases, and ECM components, collectively known as SASP, which contribute to inflammaging and immunosenescence. Inflammaging damages tissue microenvironments and reportedly plays a role in the pathogenesis of various age‐related diseases.^66^ SASP could positively influence immune system by aiding in recruitment of pre‐cancerous lesions and promoting tissue repair.^67^, ^68^, ^69^ However, it also secretes pro‐inflammatory substances such as IL‐6, IL‐8, membrane cofactor proteins, and macrophage inflammatory proteins, which could increase proliferation, angiogenesis, and inflammation thereby having unfavorable implications.^70^ SASP has been confirmed to launch spontaneous DNA damage in immune cells and higher senescence and SASP markers in various immune cells, such as monocytes/macrophages, B cells, NK cells, and T cells.^71^, ^72^ Additionally, inflammaging is triggered by increased visceral adiposity, oxidative stress, enhanced gut permeability, chronic infections, dysfunctional immune cells, and the presence of SASP.^43^, ^73^

#### 2.2.3 Altered signaling pathways

*Caenorhabditis elegans (C. elegans)*mutations in *daf‐2* gene were discovered to nearly double imaginal lifespan in 1993 by regulating switch between natural developmental processes and alternating larval diapause formation.^74^ Subsequent research revealed that senescence‐associated genes such as *daf‐2* are mammalian genes orthologues involved in insulin/IGF intracellular signaling pathway, which could be inhibited for enhanced lifespan in yeast and flies.^75^, ^76^, ^77^ Additionally, rapamycin was first found to have strong antifungal effects and was further recognized for inhibiting cell growth and modulating immune system.^78^, ^79^ Its mechanism was understood through mutants in *Saccharomyces cerevisiae* that countered its cell cycle‐arresting effects.^78^ The target of rapamycin is a multifunctional protein that merges nutrients, energy, and stress signals, manipulating autophagy, transcription, mRNA translation, and mitochondrial function, all of which are linked to increased lifespan.^80^, ^81^ Nutrient‐sensing pathways such as mammalian target of rapamycin (mTOR) pathway, may significantly affect T‐cell activation, differentiation, and overall immune responses. Moreover, increasing evidence shows that nicotinamide adenine dinucleotide (NAD^+^) levels and sirtuin activity, both crucial for cellular function, decline with age, immunosenescence, or a high‐fat diet, contributing to senescence process.^82^ In senescent T cells, activation of p38 mitogen‐activated protein kinase (MAPK) obstructs autophagy protein nine through an mTOR‐independent pathway, thereby impeding autophagic process.^83^

#### 2.2.4 Activation of immunosuppressive network

The immune system has extensive plasticity and is capable of responding and adapting to diverse kinds of microenvironmental insults. Persistent inflammation often triggers compensatory immunosuppression to defend tissues.^84^ Inflammatory mediators facilitate myelopoiesis and induce immature myeloid‐derived suppressor cells (MDSCs) generation that scale up immunosuppressive network. The immunosuppressive network comprises regulatory T (Tregs) cells, regulatory B cells, regulatory phenotypes of macrophages, dendritic, NK, type II NKT cells, and immature MDSCs.^85^ These members are able to amplify each other's suppressive functions and promote the differentiation of additional immunosuppressive cells.^86^, ^87^ Immunosuppressive cells release immunosuppressive factors such as ROS, IL‐10, transforming growth factor beta (TGF‐β), indoleamine 2,3‐dioxygenase, and arginase‐1, which inhibit immune cell function. As proof, TGF‐β exposure halts T‐cell proliferation by upregulating CDK inhibitors, inhibits T‐cell activation, disrupts helper T cell (Th) cell differentiation, reduces CD8 T‐cell cytotoxicity, and disrupts ECM architecture.^88^ To sum up, activated immunosuppressive network inhibits functions of immune cells through: (1) hindering immune cell growth and proliferation. (2) Reducing CD8 T and NK cell cytotoxicity. (3) Blocking antigen presentation and antibody production. (4) Dampening responses to inflammatory signals.

#### 2.2.5 Energy and substance metabolism disorders

Immune function relies heavily on nutritional metabolism, known as immunometabolism.^89^ Senescence disrupts basic nutrient metabolism (glucose, lipids, amino acids) in immune cells, affecting NAD^+^ metabolism, triggering inflammation as well as speeding up immunosenescence.^90^ During immunosenescence, reduced glycolytic metabolism and abnormal mitochondrial energy impair T and B‐cell activation.^91^, ^92^ NAD^+^, a coenzyme essential for cellular metabolism, suffers decline with senescence in response to decreased biosynthesis from chronic inflammation, oxidative stress, and increased depletion from DNA damage.^93^, ^94^ This reduction activates NOD‐like receptor family, pyrin domain containing 3 inflammasomes, potentially accounting for inflammatory diseases.^95^ Furthermore, proteostasis deteriorates with age, crucial for maintaining protein structure and function, impacting immune responses, particularly in T cells, accounting for protein accumulation in tissues. Furthermore, amino acid metabolism deteriorates and a wide range of proteins fail to degrade and accumulate in tissues, which affects immune responses, particularly that of T cells, ultimately leading to immunosenescence‐related diseases.^96^, ^97^

**FIGURE 2:**
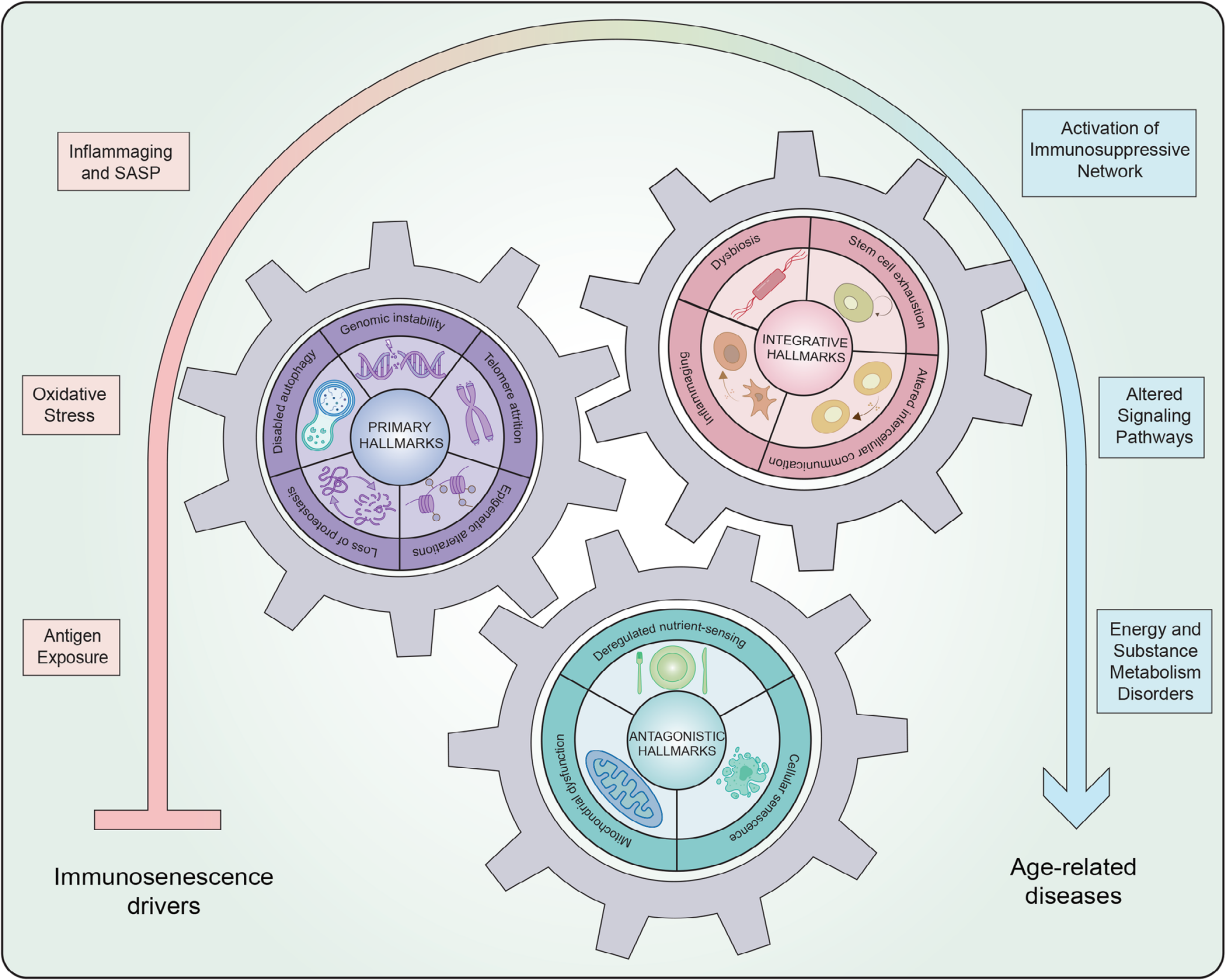
Hallmarks and molecular mechanism of immunosenescence. Hallmarks of immunosenescence include primary hallmarks (genomic instability, telomere attrition, epigenetic alterations, loss of proteostasis, and disabled macroautophagy), antagonistic hallmarks (deregulated nutrient‐sensing, mitochondrial dysfunction, and cellular senescence), integrative hallmarks (stem cell exhaustion, altered intercellular communication, inflammaging, and dysbiosis). These three clusters of hallmarks act like gears in advancing the healthy organism toward age‐related diseases in response to immunosenescence drivers. Molecular mechanisms underlying immunosenescence containing antigen exposure, and oxidative stress, inflammaging and senescence‐associated secretory phenotype, altered signaling pathways, activation of immunosuppressive network, and energy and substance metabolism disorders likewise facilitate this process.

To sum up, we will refer to these hallmarks of immunosenescence could prolong healthspan and lifespan, but they follow a certain hierarchy. The primary hallmarks (i.e., genomic instability, epigenetic alterations, loss of proteostasis, disabled macroautophagy, and telomere attrition) injure genome, epigenome, proteome, organelles, and telomeres and build up over time and initiate immunosenescence at upstream level. Further downstream, the antagonistic hallmarks (i.e., deregulated nutrient‐sensing, mitochondrial dysfunction, cellular senescence) reflect a maladaptive response to injury and play a more subtle role in the aging process. Ultimately, integrative hallmarks (i.e., stem cell exhaustion, altered intercellular communication, inflammaging, and dysbiosis) emerge when damage from primary and antagonistic hallmarks becomes irreparable, collectively leading to drive aging along with molecular mechanisms (Figure 2).

### 2.3 Immunosenescence‐associated changes in immune cells

**FIGURE 3:**
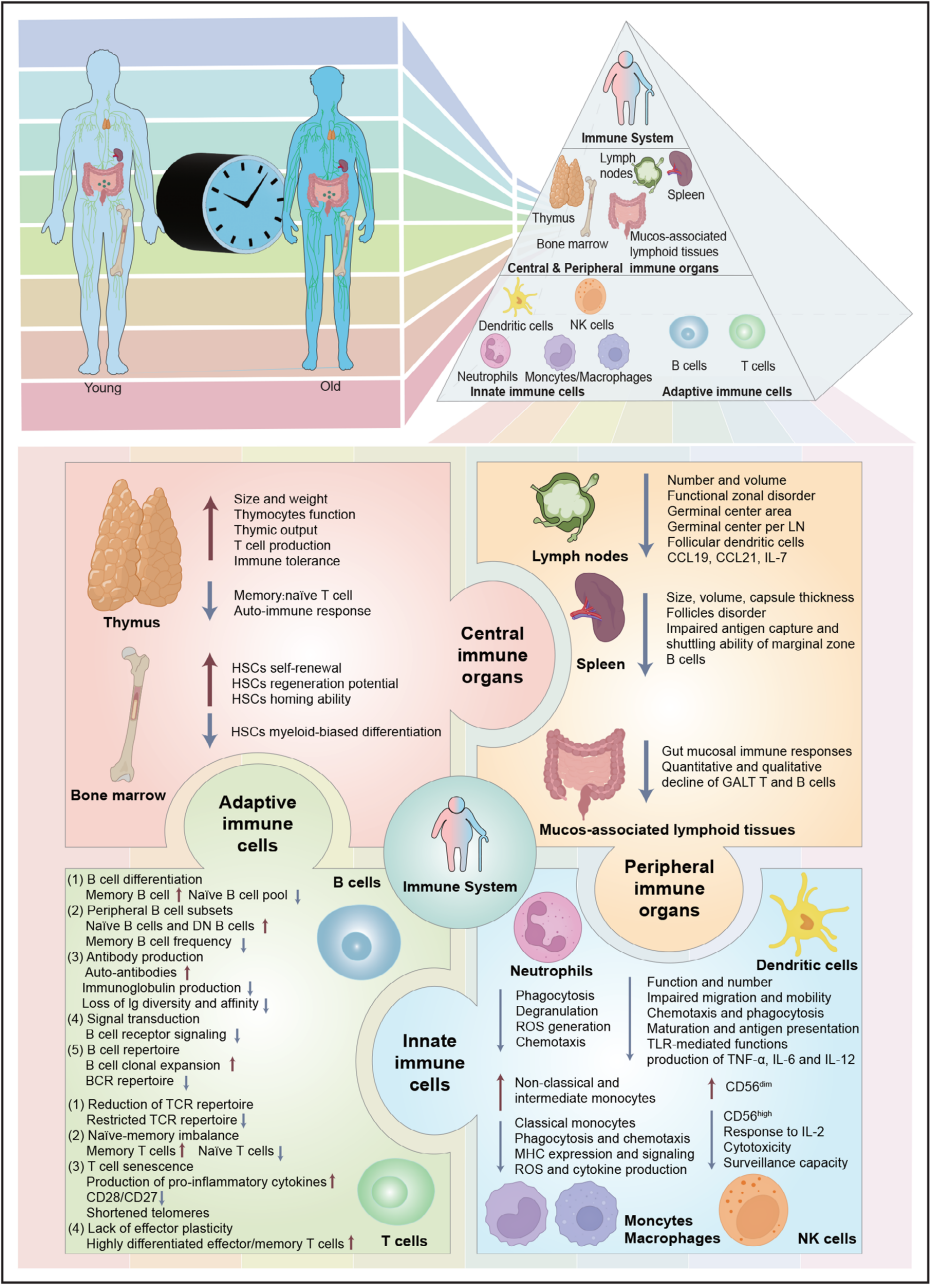
Immunosenescence‐associated changes in immune cells and organs. Immune system of human organism has a defined structural hierarchy. Over time, human organism undergoes significant changes through influence of immunosenescence. Macroscopic view of organismal senescence can be seen in its more microscopic manifestations under magnification of trigonal cone. Here, we map changes in innate immune cells (neutrophils, monocytes/macrophages, antigen‐presenting cells, and natural killer cells), adaptive immune cells (B cells and T cells), central immune organs (thymus and bone marrow), and peripheral immune organs (lymph nodes, spleen, and mucosa‐associated lymphoid tissues).

A thorough understanding of immunosenescence‐associated changes in immune system could facilitate identifying alternative treatments to improve immune responses and vaccine efficacy in old folks. Innate immunity impedes pathogenic agents through cellular and biochemical responses that are typically rapid and non‐specific. Cellular constituents of innate immunity include neutrophils, monocytes, macrophages, APCs, and NK cells. Additionally, immunosenescence inevitably contributes to heightened frequency and severity of infectious diseases and a decline in the protective benefits of vaccines in the old. The diminished vaccine effectiveness may be attributed to widespread deficiencies in B cells and T cells. Table 1 enumerates the main immunosenescence‐associated changes in immune cells. In this context, we also portray alterations linked to immunosenescence in immune cells and organs (Figure 3).

**TABLE 1:** The main immunosenescence‐associated changes in immune cells.

Cell	Phenotype	Changes	Causes	Effects	Therapeutic approach
Neutrophils	CD66b^+^/CD16^+^	Reduced response to chemotactic signals; Reduced apoptosis; Impaired signal transduction; Decreased superoxide production; Decreased MHC expression; Reduced recruitment into lipid rafts; Reduced NETs formation; Reduced adhesion and degranulation; Increased reverse transendothelial migration.	Life‐long antigenic stimulation by chronic infection; Cellular senescence.	Delayed wound healing; Inflammatory environment; Age‐related diseases; Increased mortality; Increased infections risk; Tumorigenesis.	Statins; Vitamin E; Eicosapentaenoic acid; Physical exercise.
Monocytes	Classical (CD14++CD16^−^) Intermediate (CD14^+^CD16^+^) Non‐classical (CD14^dim/low^CD16^+^)	Reduced absolute number and frequency of classical monocytes, increase of intermediate and non‐classical monocytes; Reduced phagocytosis and chemotaxis; Decreased MHC expression and signaling; Decreased ROS and cytokine production; Decreased autophagy and energy metabolism; Altered TLR expression and compromised function.	Life‐long antigenic stimulation by chronic infection; changes in myeloid progenitors; Changes in surface receptor expression; Cellular senescence.	Slow would healing; Inflammaging; Immune response against infectious diseases; Increased incidence of autoimmune disease; Tumorigenesis.	COX‐2 inhibitors; Flavonoid‐rich cocoa polyphenols; Long‐term exercise.
Macrophages		Reduced frequency in blood and bone marrow; Reduced phagocytosis of bacterial pathogens; Reduced MHC‐II expression and antigen presentation; Increased expression of COX2 and PGE2 production; Increased production of pro‐inflammatory cytokines and reduced production of anti‐inflammatory cytokines; Reduced TLR expression; Decreased autophagy; Decreased M1 polarization; Increased p38 activity.	Life‐long antigenic stimulation by chronic infection; Changes in myeloid progenitors; Changes in surface receptor expression; Cellular senescence.	Reduced capacity to phagocytose antigens; Impaired ability to respond to infections; Increased incidence of autoimmune disease; Tumorigenesis.	IL‐4 treatment; Type 2 cytokine signaling; IL‐2/CD40 therapy; p38 inhibition.
Dendritic cells	Plasmacytoid (CD11c^+^CD123^−^) Myeloid (CD11c^−^CD123^+^)	Decreased maturation and antigen presentation; Reduced phagocytosis in mDCs; Altered TLR expression and signaling in pDCs; Altered CD80 and CD86 expression. Increased production of IL‐6 and TNF‐α; Diminished IFN‐γ production; Reduced mobility; Reduced capability of CD8^+^ T‐cell activation.	Life‐long antigenic stimulation by chronic infection; Changes in myeloid progenitors; Changes in surface receptor expression; mDC and pDC constitutively activated; Reduced ability to migrate to local lymph nodes; Cellular senescence.	Impaired antigen‐presenting capacity; Impaired ability to respond to infections; Increased incidence of autoimmune disease; Decreased vaccine response; Decreased antibody secretion.	TLR agonists
NK cells	Secreting‐cytokines (CD56^high^CD16^low^) Cytotoxic (CD56^dim^CD16^+^)	Decreased fraction of CD56^high^ NK subset, expansion of cytotoxic NK subset; Decreased cytokines production of CD56^high^ NK cells; Decreased lytic capacity of CD56^dim^ NK cells; Impaired perforin degranulation; Reduced cytotoxicity; Reduced mobilization; Decreased response to IL‐2; Reduced surveillance capacity.	Life‐long antigenic stimulation by chronic viral infection; Thymic involution; Cellular senescence.	Slow wound healing; Increased mortality; Immune surveillance and tumorigenesis.	NKG2A blockade; Physical activity; Zinc supplementation; Vitamins B9 and B12.
B cells	Naïve (IgD^+^CD27^−^) IgM or unswitched memory (IgD^+^CD27^+^) Switched memory (IgD^−^CD27^+^) Double negative (IgD^−^CD27^−^)	(1) B‐cell differentiation: Reduced naive B‐cell pool; Increased Memory B cell. (2) Peripheral B‐cell subsets: Reduced number or percentages of naïve B cells, increase of double negative memory B cells; Maintained number or percentages of unswitched memory B cells; Maintained or decreased number or percentages of switched memory B cells and IgM memory B cells. (3) Antibody production: Increased auto‐antibodies; Reduced Ig production; loss of Ig diversity and affinity. (4) Signal transduction: Decreased B‐cell receptor signaling. (5) B‐cell repertoire: B‐cell clonal expansion; Restricted BCR repertoire.	Reduction in hematopoietic stem cell progenitors; Phenotypic conversion of naïve B cells into memory phenotype; Defective responses of memory CD4^+^ or CD8^+^ T cells, inability to provide help to B cells in part due to higher DUSP4 expression.	Increased susceptibility to infectious diseases; Reduced ability to respond to new pathogens and reduced protection of vaccination; Maintained immune response against well‐known antigens; Increased secretion of pro‐inflammatory cytokines.	B‐cell depletion; B‐cell rejuvenation.
T cells	Naive (CD45RA^+^/CCR7^+^/CD27^+^/CD28^+^) T_CM_ (CD45RA^−^/CCR7^+^/CD27^+^/CD28^+^) T_EM_ (CD45RA^−^/CCR7^−^/CD27^−^/CD28^−^) T_EMRA_ (CD45RA^+^/CCR7^−^/CD27^−^/CD28^−^)	(1) Reduction of TCR repertoire: Restricted TCR repertoire; Defects in TCR and co‐receptors functioning; Impaired homeostatic proliferation and decreased T‐cell repertoire. (2) Naïve‐memory imbalance: Naïve T cells decreased in number/percentages; Increased effector and memory T cells. (3) T‐cell senescence Reduced cytotoxicity; Decreased production of IL‐2; Impaired signal transduction; Dysregulation of cytoskeletal function; Defective protein glycosylation and activation; Increased secretion of pro‐inflammatory cytokines. (4) Lack of effector plasticity: Impaired, or absent, proliferation capacity; Normal or increased in number/percentages of T_CM_; T_EM_ normal or increased in number/percentages; T_EMRA_ normal or increased in number/percentages.	Reduction of hematopoietic stem cell progenitors; Defects in thymic stromal niches; Phenotypic conversion of naïve cells into memory phenotype; Reactivation of persistent virus infections.	Reduced responses to cognate antigens; Reduced responses to new antigens and neoantigens; Increased susceptibility to infections, auto‐immune disorders, chronic diseases, cardiovascular disease, and cancer. Increased inflammatory status; Decreased vaccine response; Decreased antibody secretion.	mTOR; DUSP4 and 6; VPS39 inhibition; IL‐7 administration; CD153 vaccination; Sestrin inhibition; Exercise and nutrients; Adoptive T‐cell therapy.
Thymocytes		Reduced thymocytes function	Life‐long antigenic stimulation by chronic viral infection; Cellular senescence.	Decreased thymus size and weight; Decreased thymus output; Decreased immune tolerance; Increased autoimmune response.	FOXN1 induction; Thymus reconstitution.
Hematopoietic stem cells		Reduced self‐renewal potency and regeneration potential; Increased apoptosis; Impaired homing ability; Decreased energy metabolism; Skewing of bone marrow progenitors toward the myeloid lineage; Increased p38; Increased CDC42.	Epigenetic changes; DNA damage; Telomere shorting; Metabolism alterations; Exposure to chronic age‐related inflammation.	Fewer circulating lymphocytes; Increased susceptibility to anemia; Increased pyogenic bacterial infection.	p38 inhibition; CDC42 inhibition; HSCs transplant.
Myeloid‐derived suppressor cells		Increased frequency in the circulation of very old (>80 years) or frail people.	Infections and cancer.	Lower capacity to clear the infection.	

Abbreviations: BCR, B‐cell receptor; CDC42, cell division control protein 42; COX, cyclooxygenase; DUSP, dual‐specificity phosphatase; FOXN1, Forkhead box N1; HSCs, hematopoietic stem cells; IFN, interferon; Ig, immunoglobulin; IL, interleukin; M1, classical activated macrophages; mDCs, myeloid DCs; MHC, major histocompatibility complex; mTOR, mammalian target of rapamycin; NETs, neutrophil extracellular traps; NK, natural killer; NKG2A, killer cell lectin‐like receptor C1; pDCs, plasmacytoid DCs; PGE2, prostaglandin E2; ROS, reactive oxygen species; T_CM_, central memory T cell; T_EM_, effector memory T cell; T_EMRA_, terminally differentiated effector memory T cell; TCR, T‐cell receptor; TLR, Toll‐like receptor; TNF, tumor necrosis factor; VPS39, VPS39 subunit of HOPS complex.

#### 2.3.1 Innate immune cells

*Neutrophils*: Unlike other cell populations, neutrophils do not exhibit a distinct senescence phenotype, thereby underscoring the need for a comprehensive characterization of their role in the immunosenescence process.^98^ Microbicidal activities of neutrophils in older adults have been reported to be massively curtailed, primarily due to hampered phagocytosis, degranulation, and ROS generation.^99^ Furthermore, migration of neutrophils reacting to chemotactic stimuli is similarly diminished in the aged, namely evoking defective chemotaxis. This impairment affects capacity of neutrophils to reach injury sites, alters their distribution, and further hinders recognition and clearance of pathogens.^100^ In addition, reduced chemotactic response may exacerbate tissue damage and inflammation due to neutrophil‐released proteases, such as neutrophil elastase, which facilitates migration in tissues.^101^

*Monocytes/macrophages*: Monocytes take part in generating anti‐inflammatory cytokines and lipidic mediators and empower them to participate in wound healing and tissue restructuring.^102^ These cells are heterogeneous and highly adaptive, responding to pathogens and cellular debris, and simultaneously able to engage with adaptive immune system. Three distinct subpopulations of monocytes could be differentiated based on expression of surface molecules: (1) classical CD14^++^CD16^−^ monocytes; (2) intermediate CD14^+^CD16^+^ monocytes; and (3) non‐classical CD14^dim/low^CD16^+^ monocytes. It is further noted that there is a transition from classical monocytes to intermediate monocytes and subsequently to non‐classical monocytes. These subsets exhibit immunosenescence‐like phenotypes characterized by shortened telomeres, eliciting inflammaging states.^103^, ^104^ Consequently, it is concluded that advancing immunosenescence seems to be correlated with elevation in non‐classical monocytes and intermediate monocytes.^104^

*APCs*: Dendritic cells (DCs), the most crucial APCs, are specialized in uptake, processing, and presentation of antigens to T cells. Human DCs could be categorized as plasmacytoid DCs (pDCs) and myeloid DCs (mDCs), each exhibiting distinct functional activities. pDCs are primarily involved in interferon‐α/β production against bacterial and viral infections, whereas mDCs are responsible for IL‐12 production as well as inducing Th1 and cytotoxic T lymphocyte (CTL) responses.^105^ Frustratingly, immunosenescent DCs could damage migration and phagocytosis, possessing a diminished or delayed potential to migrate to lymphoid organs and peripheral tissues, and eventually bring about chilly antigen presentation.^106^ Additionally, a spectrum of TLRs is differentially expressed and functionally handicapped in the elderly, resulting in altered TLR‐mediated immune responses. Elderly mDCs and pDCs are pronounced dampened in their ability to participate in TLR‐stimulated secretion of TNF‐α, IL‐6, and IL‐12, attending to impaired vaccination responses in older people.^107^

*NK cells*: NK cells are dissected to be guardians of innate immunity, playing an instrumental role in defense against viral infections and tumor targets. They contribute to immunosurveillance by releasing soluble factors and cell‐to‐cell contact‐mediated interactions. The link between NK cell deficiency and substantially increased viral infection incidence unravels that NK cells are critical in the early clearance of virus and prior to cancer control, preceding activation of adaptive immune responses.^108^ NK cells could also clear cells that multiply with immunosenescence and interplay with macrophages and DCs to modulate their activation and functions.^109^ There are distinguished subpopulations of NK cells based on differential expression of phenotypic and functional markers: (1) CD56^high^CD16^low^ NK cells which are more immature, and (2) CD56^dim^CD16^+^ NK cells which consist of mature NK cells.^110^ Immunosenescence induces a redistribution of NK cell subpopulations, characterized by an enlarged proportion of mature NK cells and a notable decline in immature NK cell subpopulations. More significantly, immunosenescence also aggravates variations in NK cell functions, as represented by disrupted NK cell proliferation in response to IL‐2 stimulation.^111^ Ultimately, while immunosenescence does not alter overall NK cell cytotoxicity attributed to augmented frequency of mature NK cells, it does result in impaired NK cell cytotoxicity in individual cells owing to diminished activation receptor expression.^112^

#### 2.3.2 Adaptive immune cells

*B cells*: Impact of immunosenescence on B cells could be summarized in the following five dimensions: (1) B‐cell differentiation. A study conducted by Nunez et al. examining a human population found no obvious changes in pro‐B cells, pre‐B cells, and immature B‐cell frequencies among individuals aged 24–88 years.^113^ On the contrary, linear regression analysis in bone marrow samples from several cancer or non‐cancer patients revealed that B lineage precursors waddled down with the onset of immunosenescence.^114^ It has been proposed that immunosenescence impedes B‐cell differentiation and output of mature B cells in bone marrow. (2) Peripheral B‐cell subsets. Both percentage and absolute number of B cells have been documented to drop off dramatically with age, and proportions of disparate B‐cell subpopulations also shift in humans.^115^ Four main subpopulations could be identified by expression of anti‐CD19, anti‐CD27, and immunoglobulin (Ig) D antibodies: naïve (IgD^+^CD27^−^) B cells, IgM or unswitched memory (IgD^+^CD27^+^) B cells, switched memory (IgD^−^CD27^+^) B cells and double negative (IgD^−^CD27^−^) B cells. Immunosenescence decreases proportion of switched memory B cells and significantly increases proportion of naïve and double‐negative B cells. Even so, it manifests no change in IgM memory B cells. However, additional studies have illuminated reduced IgM memory B cells in older adults, which is postulated to hasten susceptibility to pneumococcal infections.^114^ (3) Antibody production. Compared to younger individuals, mitogen‐stimulated B cells in old folks display reductive levels of E47 and activation‐induced cytidine deaminase expression and secrete less amount of IgG. Furthermore, reduced influenza vaccine responses in older adults could be partly attributed to specific serum antibodies, switching of memory B cells, and long‐lived plasma cells.^116^, ^117^ It is confirmed that capacity to elicit vaccine‐specific antibody responses is inversely related to serum TNF‐α level.^118^ The outgrowth is that human unstimulated B cells in the aged secrete greater levels of TNF‐α than B cells in younger adults. (4) Signal transduction. B‐cell immunosenescence could produce a harmful effect on cell activation and signaling. Moreover, a preclinical assay underlined that calcium/calmodulin‐dependent protein kinase type IV (CaMKIV) is phosphorylated after in vitro irritation of influenza‐vaccinated peripheral blood mononuclear cells from young adults.^119^ CaMKIV is a constituent of calcium/CaMK family, which may guide Ca^2+^‐dependent cellular functions and inflammatory responses through TNF‐α generation. It was found that phosphorylated CaMKIV levels were negatively proportional to serum antibody titers 3 days after vaccination, indicating that CaMKIV may play a part in ordering antibody responses to influenza vaccines. (5) B‐cell repertoire. Some researchers have examined changes in antibody repertoire with immunosenescence. Using Ig heavy chain complementarity‐determining region 3 spectrometry to analyze DNA samples of peripheral blood from 19 to 94‐year‐old ascertained a considerable paucity of diversity in older individuals’ antibody repertoire, coupled with oligoclonal amplification, the absence of diversity positively correlated with frailty.^120^ Over‐expansion of plasma cells recruits unspecified monoclonal gammopathy and additional monoclonal B‐cell expansions, which are closely tied to immunosenescence.^121^ To provide inimitable insights, another study using spectra‐type analysis and next‐generation sequencing exhibited that without antigenic challenge, B‐cell complexes in older adults displayed non‐specific clonal expansion.^122^ Thus, we arrive at a conclusion that lack of specific B‐cell diversity has an intimate association with impaired health.

*T cells*: Immunosenescence could affect diversiform spheres of T‐cell functions, involving their clonality or specificity functions, degree of chronological differentiation, proliferative capacity, and deviations in their activity. The straitened circumstances of immunosenescence on time‐dependent deterioration of T lymphocytes could be summarized in the following four aspects: (1) reduction of T‐cell receptor (TCR) repertoire. TCR repertoire also varies with immunosenescence related to a vast majority of elements including thymic involution and so on. Shreds of evidence from longitudinal and cross‐sectional population studies implied that both endogenous and exogenous T‐cell factors may aggravate a hindrance in TCR diversity to scuttle immune functions and induct an over‐expansion of (autoantigens or persistent viruses that encode antigen‐specific T cells.^123^ (2) Naïve‐memory imbalance. Naïve cell population shrinks with immunosenescence in response to the buildup of highly differentiated memory cells, which may in turn, reflect depletion of stem cell‐like populations in T‐cell repertoire. Interestingly, Mold and colleagues take the attitude that CD8^+^ naïve T cells revealed more recessionary homologous proliferation in vivo compared with CD4^+^ naïve T cells.^124^ Naïve T cells will differentiate toward memory T cells as a result of lifelong antigenic stimulations. In a nutshell, the available data are suggestive of increases in memory, of which some are antigenically inexperienced T cells and reductions in naïve cell pool arising from thymic involution, sustained antigenic irritations, and inflammatory milieus. (3) T‐cell senescence. T cells obtain a senescent phenotype through continued antigenic stimulation, especially chronic virus infections which limits T‐cell responses.^125^ T‐cell immunosenescence could secrete substantial pro‐inflammatory factors such as TNFs and osteopontin, which are evocative of SASP. T‐cell immunosenescence also exhibits a sea of other characteristics, for instance, poor telomerase activity, telomere shortening, DNA damage evidence, and β‐galactosides. Furthermore, immunosenescent T cells de‐express costimulatory molecules CD27 and CD28 as well as up‐regulate the expression of terminal differentiation markers.^126^ Collectively, immunologically weakened but pro‐inflammatory T cells gradually multiply and accumulate along with immunosenescence. (4) Lack of effector plasticity. Differentiated CD4^+^ T lymphocytes are commonly categorized as different subtypes, which have recently been reassessed, suggesting that the Th lineage forms a continuously polarized phenotype determined by antigenic challenge.^127^ Immunosenescent T cell shed their quiescent state and enter a stage of terminal differentiation, which may execute deviations in T‐cell lineage commitment, resulting in a lack of plasticity and a decline in immune system's ability to address de novo antigenic challenges.

### 2.4 Immunosenescence‐associated changes in immune organs

#### 2.4.1 Central immune organs

*Thymus*: Thymus is main organ in charge of generating a diversified but selective population of T‐cell repertoire. Meanwhile, thymus involution is considered among the optimal‐documented hallmarks in human immune system.^128^ Thymic involution begins in childhood and climaxes in adolescence. Subsequently, the adipose tissue gradually starts to replace cortex and medulla of thymus.^129^ Immunosenescence‐related degeneration of human thymus is a breakdown of tissue architecture, shrinkage in thymus size and mass, and a drop in thymocyte numbers. Thymic degradation due to immunosenescence may be a direct reason for decreasing naïve T‐cell generation, compensatory amplification of memory T‐cell clones, as well as attenuated diversity of peripheral T‐cell pool. Thymic involution could also result in lower T‐cell functional activity, leading to decreased immunity with potentially defective immune tolerance and enhanced autoimmune responses.^130^ Ultimately, developing an understanding to revitalize thymic functions and T‐cell production offers an alternative strategy for immunosenescence interventions.

*Bone marrow*: Immunosenescence arises from senescence of hematopoietic stem cells (HSCs) for virtually all of its cells originate from HSCs. HSC microenvironment in bone marrow provides a balance that assures hematopoiesis by managing the multiplication, self‐renewal, polarization, and relocation of HSCs and progenitor cells in a stable state, as well as in response to contingencies.^131^ Most HSCs are in a stationary or dormant state, beneficial for preserving their functions and juvenile stemness, whereas multiplication contributes to depletion. However, immunosenescent bone marrow has more myeloid‐dominant HSCs but produces fewer mature blood cells per HSC. Meanwhile, competitive transplantation trials have proven that immunosenescent HSCs could inhibit self‐renewal, regenerative potential, and homing capacity.^132^ It has been performed that immunosenescence of endothelial cells is competent to actuate hematopoietic aging phenotypes among young HSCs.^133^ Insights into bone marrow microenvironment's role in underpinning HSC functions may be rewarding in managing functional hematopoietic decline linked to immunosenescence.

#### 2.4.2 Peripheral immune organs

*Lymph nodes (LNs)*: Human body has 300−500 LNs with a total weight of about 100 g.^134^ The structure and composition of the functional areas of the LNs become progressively disorganized with the accumulation of degenerative alterations. Additionally, quantity of lymphoid tissues in cortex and medulla of LNs descends, along with number and diameter of lymphoid follicular germinal centers decrease. Moreover, follicular DC cells shrink in number, LN homeostasis is progressively disrupted, and levels of relevant chemokines (e.g., CCL‐19, CCL‐21, and IL‐7) decrease.^135^, ^136^ Ultimately, these structural and functional deficits in the LNs triggered by immunosenescence eventually result in scarce T and B cells’ cellular homeostasis, the diminished ability of immune cells to recognize antigens, and weakened immune responses.^7^ Understanding the reasons for these stromal and lymphoid microenvironment shifts in LNs that spur the immunosenescence‐related dysfunction of immune system could optimize long‐term immune responses and effectiveness of vaccines in the elderly.

*Spleen*: Immunosenescence decreases diameter and bulk of human spleen. Specifically, thickness of splenic capsule could also be affected by age: from birth to puberty, it progresses to its zenith and then begins slowly thin. What's more, follicles become abnormal, and somatic mutations in the splenic germinal centers lessen with age.^137^ Marginal zone B cells are located in spleen to trap antigens and immune complexes in blood and convey them to follicular DCs in B‐cell follicles. Interestingly, it has been concluded that marginal zone B cells have an impaired capacity to trap antigens and pass between the follicular and marginal zones in spleen of old mice.^138^

*Mucosa‐associated lymphoid tissues (MALTs)*: MALTs, differing from other immune organs throughout human body, suffer virtually no roadblock to immune functions during immunosenescence and play an essential part in elderly immunity.^139^ The obligatory role of commensal bacteria in advance of maximal mucosal immune system function has been substantiated and its components with potent immune‐activating features are implicated in pleiotropic diseases.^140^ During immunosenescence, gut‐associated lymphoid tissues (GALTs) immune response is modified. An immunosenescence‐induced decline in number and mass of GALT T and B cells along with their subpopulations largely account for suppressed intestinal mucosal immune responses.^141^ The implications of immunosenescence on the composition, diversity, and functions of the intestinal flora, as well as the persistent chronic inflammatory stimuli caused by aging, lead to immunosenescence of the gastrointestinal mucosal lymphoid tissues at length. Modulation of microflora components with probiotics and prebiotics puts forward an alternative strategy to develop mucosal immunity in parallel with preventing and treating a slice of diseases.^142^

The hallmarks, molecular mechanisms, and ramifications of immunosenescence are intricately interconnected. Various forms of molecular damage, instigated by immunosenescent hallmarks and mechanisms, affect organelles, thereby altering cellular function and even cell fatality. These alterations subsequently influence organ homeostasis, resulting in significant repercussions for entire organism.

## 3 IMMUNOSENESCENCE AND TUMORIGENESIS

Older people are at higher risk for tumorigenesis. Individuals over 60 are more than twice as likely to suffer from progressive and invasive cancers than younger people, which determines that cancer is a disease of immunosenescence.^143^, ^144^ Countless researchers have made out that immunosenescence may also be imperative tumorigenesis despite the accumulation of genetic mutations. The assay by Palmer et al. concluded that the shift in immune system may play a major part in development of tumors from an analysis of the age distribution of 100 different tumors.^145^

Immune system plays a double‐edged sword effect because it provides anti‐tumor activity despite its close connection to cancer onset and progression.^146^ In young and healthy people, immune system could prevent proliferation of malignant tissues and remove damaged cells, while decreased immune system functions could directly contribute to development of SASP, inflammation, and tumors. This physiopathological network handicaps innate immune and adaptive immune functions, bolstering recruitment of immune cells and orienting them toward immunosuppressive functions and susceptibility to cancer development.^147^ Immune system could remove those abnormal cells from human body, blocking tumor cells from proliferating uncontrollably. Immune system wins the war against tumor growth mainly through three different ways: (1) exterminate or inhibit viral infections to prevent the host from virus‐induced tumors. (2) Suitably eliminate pathogens to protect the inflammatory milieus from establishing conducive to tumorigenesis. (3) Specifically recognize and throttle tumor cells based on the expression of tumor‐specific antigens and cellular stress‐inducing molecules.^148^ Immunosenescence affects responsiveness to extrinsic antigens and rationally reduces anti‐tumor immune defenses in older adults.^149^ According to the aforementioned views, it is imaginable that immunosenescence is an additional factor that further contributes to the tumor escape mechanism.

Tumor cells could also escape from the immune system in several cases. Immunocytes, ECM, blood vessels, adipocytes, and sundry molecules have been noted to sustain tumor development with architectural, irrigation, and energy support. Together, all these components that concomitant and facilitate tumor viability and proliferation are defined as TIME.^150^, ^151^ Immunosenescence greatly affects TIME resulting in promoting tumorigenesis and progression through the following mechanisms: (1) immunosenescence impairs the integrity of ECM and facilitates tumor development. ECM is a physical barrier, anchoring point, and cell migration pathway, whose integrity is significantly parallel with age.^152^ (2) In immunosenescent TIME, stromal cells are disorganized and dislocated thereby incurring tumorigenesis and progression. (3) Immunosenescent TIME also harbors immunosuppressive cell infiltration such as MDSCs which reduces tumor clearance.^85^ The accumulation of immunosuppressive N2 neutrophils and M2 macrophages could also induce further immunosuppression. However, the cytotoxic activity of immunosenescent macrophages, DCs, NK cells, and effector T cells is substantially diminished owing to tumor escape and progression. Concurrently, MDSCs may promote tumor development by altering ECM composition.^153^ (4) Accumulated secretion of SASP factors (e.g., IL‐6, IL‐8, and IL‐10) could also promote tumor progression and metastasis. In addition, studies have underlined that numerous molecules with SASP characteristics are ECM‐degrading enzymes, which could remodel ECM to establish a favorable milieu for cancer cell invasion, spread, and immune cell trafficking.^154^, ^155^ Likewise, interactions between secretions connected with SASP and immunosuppressed populations could bolster MDSCs infiltration in tumor locations.

**FIGURE 4:**
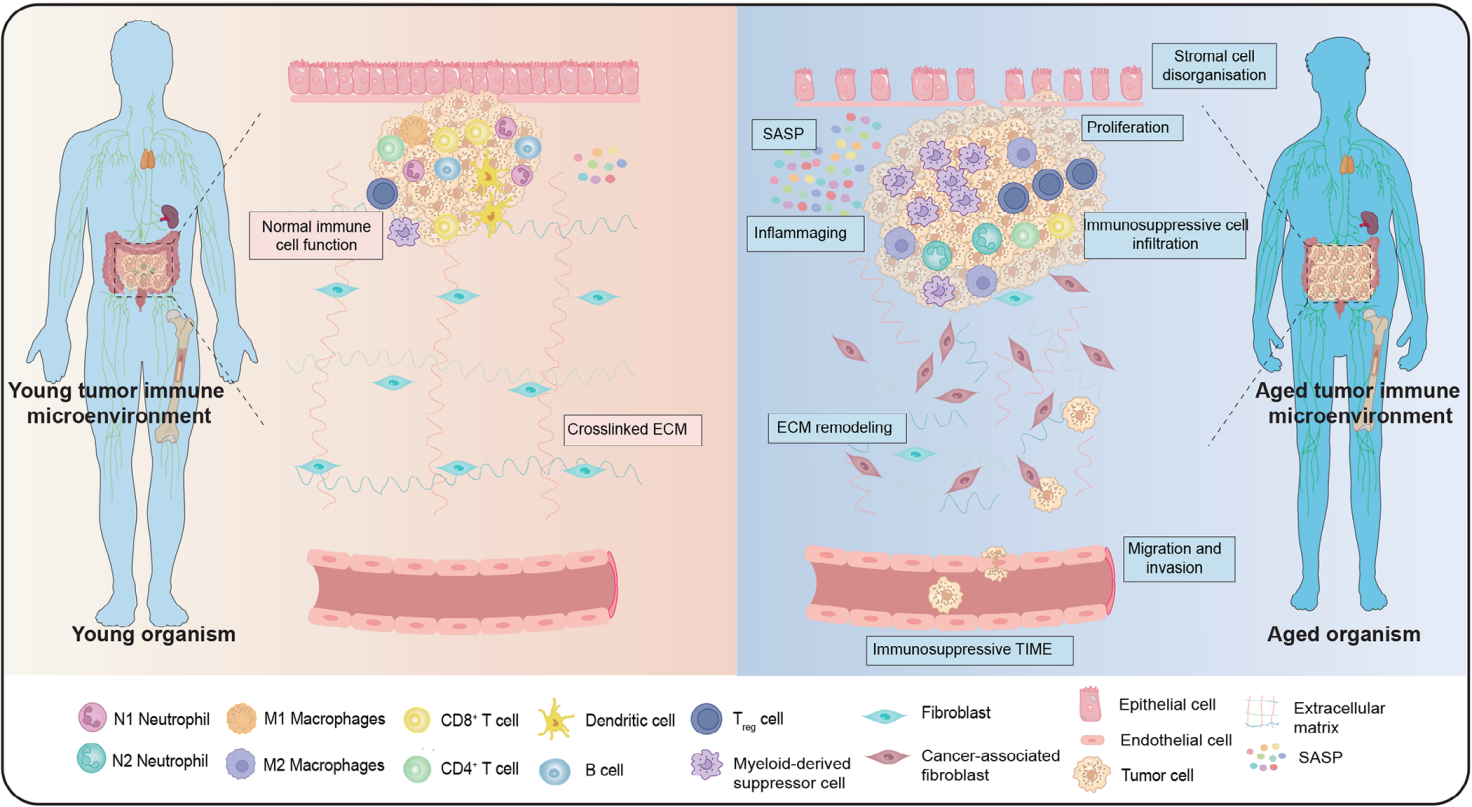
Impact of immunosenescence on tumorigenesis. The shift in immune system due to immunosenescence plays a major part in development of tumors. Immunosenescence impairs the integrity of extracellular matrix and stromal cells thereby incurring tumorigenesis. Immunosenescent tumor immune microenvironment also harbors immunosuppressive cell infiltration and secretion of senescence‐associated secretory phenotype factors to promote tumor progression. Together, all these components impacted by immunosenescence could facilitate tumor viability and proliferation.

Taken together, it seems that inflammaging, SASP, ECM remodeling, and immunosuppressive TIME are interlinked with immunosenescent tumorigenesis (Figure 4). Consequently, it warrants further illustration of changes in immune cell subpopulations associated with immunosenescence in cancer assay. Since oncogenesis is an incredibly complicated occurrence, it is challenging to recognize the exact role of immunosenescence. Further research and sustained efforts in this area will assist us in recovering a well‐functioning immune system in older individuals and provide cutting‐edge avenues for eradicating tumors.

## 4 TREATMENT STRATEGIES TARGETING IMMUNOSENESCENCE

Since an immunosenescent immune system could significantly drive immunosenescence of other tissues, reactivation of effects and functions in immune cells and organs has huge potential for counteracting immunosenescence to enhance vaccination response. Given that an immunosenescent immune system could substantially contribute to immunosenescence of other tissues, reactivation of functions and effects in immune cells and organs holds considerable potential for mitigating immunosenescence, thereby enhancing immune responses. In this section, we outline major anti‐immunosenescence interventions described in the text (Table 2). New therapies targeting immune hallmarks, molecular mechanisms, cells, and organs in older adults are burgeoning, many of which are showing encouraging pre‐clinical outcomes.

**TABLE 2:** Major anti‐immunosenescence interventions described in the text.

Intervention category	Treatment	Target of process	Example drugs	Major effects	References
Pharmacological intervention	NRTI	RTE	Lamivudine	Reduce IFN‐I activation and inflammaging; Diminish IFN‐I responses in SIRT6‐deficient cells; Enhance health and lifespan in SIRT6‐knockout mice.	^149^
	OGG1	Oxidative DNA damage repair	TH10785	Enables protein to completely cleave damaged DNA strand; Increase overall repair of oxidative DNA damage.	^156^
	NAD^+^ precursors	NAD^+^ metabolism	NMN	Stabilize telomeres; Decrease DNA damage; Improves liver fibrosis in mouse model.	^157^
	NAD^+^ precursors	NAD^+^ metabolism	NR	Specifically raise mitochondrial NAD^+^ levels.	^158^
	Senolytic	Tyrosine kinase	Dasatinib + quercetin	Eradicate senescent adipocyte progenitors and endothelial cells.	^159^
	Senolytic	BCL‐2, BCL‐XL, and BCL‐W	Navitoclax	Compete for inhibitory binding slots.	^160^
	Senolytic	FOXO4‐p53	FOXO4‐DRI	Inhibit p53 axis.	^161^
	Mitochondrial electron transport chain inhibitor	Mitochondrial respiration	Metformin	Stimulate AMPK to guide cellular metabolism; Suppress mTOR pathway.	^162^, ^163^
	AMPK activator	AMPK pathway		Inhibit mitochondrial electron transport chain.	
	Mitophagy inducers	–	Urolithin A	Boost insulin sensitivity in mice; Extend lifespan in *C. elegans* and aged mice.	^164^, ^165^
	mTOR inhibitor	mTOR	Rapamycin	Prolong lifespan of model organisms; Reduce CD8^+^ T‐cell immunosenescence.	^166^, ^167^
	p38 MAPK inhibitor	p38 MAPK pathway	Losmapimod	Block monocyte‐derived COX‐2‐driven inflammation; Weaken downstream inflammatory processes; Accelerate inflammatory remission.	^168^
	p38 MAPK inhibitor	p38 MAPK pathway	PD169316	Boost proteasome activity.	^169^
	Cell division control	CDC42	Statins	Amend neutrophil chemotaxis; Diminish neutrophil extracellular trap lesions.	^170^
Nutrient	Micronutrients	–	Vitamin C	Reduce oxidative stress; Modulate Inflammaging; Support function of neutrophils, monocytes, and macrophages; Promote antibody production.	^171^
	Nutraceuticals	–	n‐3 PUFAs	Inhibit formation of eicosanoids; Inhibit synthesis of pro‐inflammatory cytokines, chemokines, adhesion molecules resulting in anti‐inflammatory properties; Impact on cytokines signaling.	^172^, ^173^
	Probiotic	–	*Lactobacilli*	Boost NK cell responses; Reduce systemic inflammaging.	^174^
	Micronutrients	–	Vitamin E	Improve chemotaxis.	^175^
	Nutraceuticals	–	EPA	Reduce ROS bursts.	^175^
	Micronutrients	–	Vitamins B9 and B12	Scale up number and activity of NK cells.	^176^
Lifestyle intervention	Mediterranean diet	–	–	Bolsters inflammation and metabolic disorders.	^177^
	Calorie restriction	mTOR, sirtuins, etc.		Down‐regulate pro‐inflammatory molecules related to inflammaging and shortened lifespan	^178^
	Exercise	β‐2 adrenergic receptors	–	Decrease blood inflammatory cytokines levels; Augment innate immune responses; Decrease exhausted T‐cell number; Increase T‐cell proliferation.	^179^
Natural compounds	Sirtuin activator	SIRT1	Resveratrol	Facilitate production of immunomodulatory and anti‐inflammatory cytokines; Augment number of Tregs.	^180^
	IGF‐1 signaling inhibitor	IGF‐1 signaling	Spermidine	Induces autophagy; Lengthen lifespan in mice; Attenuate B‐cell senescence in mice.	^181^, ^182^
	Polyphenols	–	Flavonoid‐rich cocoa polyphenols	Reduce expression of adhesion molecules and pro‐inflammatory markers on monocytes.	^170^
Stem cell therapy	Yamanaka factors	Cellular reprogramming	*Oct4*, *Sox2*, *Klf4*, and *c‐Myc*	Remodel a stem cell niche; Induce stem cell activation and proliferation; Accelerate muscle regeneration.	^183^
	NF‐κB inhibitor	‒	A lentivirus expressing dominant‐negative IκBα	Increase mouse lifespan.	^184^, ^185^
	Engineered stem cell	FOXO3	FOXO3‐engineered Human ESC‐derived vascular cells	Exhibit delayed senescence, greater oxidative stress, and tumor transformation resistance.	^186^
Gene therapy	Lentiviral vectors	*KAT7*	Lentiviral vectors encoding Cas9/sg‐*Kat7*	Alleviate liver senescence; Extend health span and life span of mice.	^187^
	AAV9‐*Tert* treatment	Telomerase *Tert* gene	AAV expressing *TERT*	Rescues aplastic anemia and mouse survival.	^188^
	AAV8‐FGF21/αKlotho/TGF‐βR2	–	AAV8‐FGF21/αKlotho/TGF‐βR2	Synergistic benefits in addressing senescence‐related organ issues and disorganized ECM.	^189^
	AAV9‐ABE/sgRNA	*LMNA* gene	AAV9‐ABE/sgRNA	Correct pathogenic allele; Ameliorate RNA mis‐splicing; Reduce progerin protein in various tissues.	^190^
Cytokine therapy	IL‐4 therapy	IL‐4‐STAT6 pathway	IL‐4	Prevent macrophage senescence; Improve healthy lifespan in aged mice.	^191^

*Note*: Symbol “‒” denotes no results available.

Abbreviations: AAV, adeno‐associated virus; ABE, adenine base editor; AMPK, adenosine 5ʹ‐monophosphate‐activated protein kinase; BCL‐2, B‐cell lymphoma‐2; BCL‐W, BCL‐2‐like protein 2; BCL‐XL, B‐cell lymphoma‐extra‐large; *C. elegans*, *Caenorhabditis elegans*; CDC42, cell division control protein 42; COX, cyclooxygenase; DRI, D‐Retro‐Inverso; ECM, extracellular matrix; EPA, eicosapentaenoic acid; ESC, embryonic stem cells; FGF21, fibroblast growth factor 21; FOXO4, Forkhead box O4; IFN, interferon; IGF‐1, insulin growth factor‐1; IL, interleukin; LMNA, lamin A; MAPK, mitogen‐activated protein kinase; mTOR, mammalian target of rapamycin; NAD^+^, nicotinamide adenine dinucleotide; NF‐κB, nuclear factor‐κB; NK, natural killer; NMN, nicotinamide mononucleotide; NR, nicotinamide riboside; NRTI, nucleoside reverse transcriptase inhibitor; n‐3 PUFAs, omega‐3 polyunsaturated fatty acids; OGG1, 8‐oxoguanine DNA glycosylase 1; ROS, reactive oxygen species; RTEs, retrotransposable elements; SIRT6, sirtuin 6; TGF‐βR2, transforming growth factor beta receptor 2; Tregs, regulatory T cells.

### 4.1 Targeting hallmarks and molecular mechanisms of immunosenescence

#### 4.1.1 Repression of genomic instability and targeting immune epigenetics

*Repression of genomic instability*: Retrotransposable elements are prime contributors to genomic instability, epigenetic alterations, and cellular senescence during immunosenescence.^192^ Retrotransposable elements have also been implicated in activating and triggering IFN‐I‐mediated inflammation, serving as epigenetic drivers of inflammaging. Admittedly, the IFN‐I response is a phenotype of late senescence and contributes to the maintenance of SASP. Thereby, treating aged mice with lamivudine, a nucleoside reverse transcriptase inhibitor, reduced IFN‐I activation and inflammaging.^193^ Such drugs could even diminish IFN‐I responses in SIRT6‐deficient cells and notably enhance health and lifespan in SIRT6‐knockout mice, which grossly exhibit abbreviated lifespans, growth delays, and high type 1 long interspersed nuclear element activities.^194^ What's more, increased repair of oxidative DNA lesions through small molecule activation of 8‐oxoguanine DNA glycosylase 1 might show therapeutic effects on immunosenescence.^156^ Likewise, nicotinamide mononucleotide supplementation boosts NAD^+^, stabilizes telomeres, decreases DNA damage, and improves liver fibrosis in a mouse model.^157^ The connection between telomere dynamics and organismal senescence has been thoroughly studied for the sake of optimal therapeutic approaches.

*Targeting immune epigenetics*: Senescence leads to significant epigenetic alterations and buildup of epimutations as a consequence of histone modifications (global DNA hypomethylation and gene‐specific DNA hypermethylation). Epigenetic clocks using DNA methylation could predict age and mortality risk and assess lifespan‐extending interventions.^195^ In addition, the most effective in vitro method to counteract epigenetic senescence involves Yamanaka factors (*Oct4*, *Sox2*, *Klf4*, and *c‐Myc*), which reset epigenetic clock by converting somatic cells into pluripotent stem cells.^196^ Wang et al. used human mesenchymal precursor cells with mutations causing two types of premature aging syndrome to perform a genome‐wide CRISPR‐Cas9 screen, identifying histone acetyltransferase gene *KAT7* as an instrumental driver of senescence. Inactivating *KAT7* in human stem cells reduced histone H3 lysine 14 acetylation, suppressed p15^INK4b^ transcription, and mitigated cellular senescence. Additionally, intravenous lentiviral vectors encoding Cas9/sg‐*Kat7* delivery could reduce hepatocyte senescence, slower liver aging, and extend lifespan in both naturally and prematurely aged mice.^187^ CRISPR‐Cas9 genetic screening effectively pinpoints senescence genes, offering potential targets for senescence therapies.

#### 4.1.2 Eliminate senescent cells and regulate immune metabolism

*Eliminate senescent cells*: Senescent cells are engaged in cancer by persistently secreting low‐level pro‐inflammatory and tissue necrosis factors in large quantities.^197^ Selectively eliminating senescent cells could preclude harmful implications of immunosenescent cells without damaging healthy cell populations.^159^ Both tyrosine kinase inhibitor dasatinib and flavonoid quercetin exhibit their ability to eradicate senescent adipocyte progenitors and endothelial cells in vivo and in vitro.^160^ These drugs administered to old mice ameliorate burden on senescent cells and immunosenescent pathologies. Navitoclax and its analogs trigger apoptosis by selectively eliminating senescent cells in competing for inhibitory binding slots of BCL‐2, BCL‐XL, and BCL‐W.^161^ Besides, D‐Retro‐Inverso peptide, a Forkhead box O4 (FOXO4) isoform that primes apoptosis of senescent cells by inhibiting p53 axis, has been exploited.^198^ Although most of data on such drugs are derived from zoological models, one highly promising human trial has also been launched to assess beneficial effects of integrating small‐molecule drugs with vaccines.^199^ Unlocking the activity of human body's immune system against senescent cells is the realm of potential therapy. Monalizumab, an anti‐NKG2A monoclonal antibody, has been proven to have superior anti‐tumor ability by enabling NK and CD8^+^ T cells to throttle tumor cells that express inhibitory receptor human leukocyte antigen (HLA)‐E.^200^, ^201^ More recently, natural and synthetic therapeutic agents, as well as multiple immunotherapies have been utilized in senescent cell clearance.^202^

*Regulate immune metabolism*: A few compounds and drugs are available to orchestrate metabolism of cells in TIME. Metformin, a drug administrated in type 2 diabetes typically, stimulates adenosine 5′‐monophosphate‐activated protein kinase to guide cellular metabolism and suppress mTOR pathway, which disrupts nutrient supply to tumor cells and enhances immune cell functions in TIME.^162^ A small clinical trial combined metformin, recombinant human growth hormone, and dehydroepiandrosterone to reverse epigenetic age of healthy older adults, resulting in thymic regeneration, immune changes, and increased life expectancy.^203^ Moreover, resveratrol affects immune cells such as macrophages, T cells, B cells, and NK cells, facilitates production of immunomodulatory and anti‐inflammatory cytokines, and augments number of Tregs, thus exerting an anti‐inflammatory function.^180^ Similarly, spermidine, an endogenous metabolite that induces autophagy, lengthens lifespan and attenuates B‐cell senescence in mice.^181^, ^182^ Additionally, in TIME, 2‐deoxy‐D‐glucose, a glucose analog, effectively represses glycolysis, a critical metabolic pathway essential for energy production in cancer cells. 2‐Deoxy‐D‐glucose could modify metabolic paradigm of TIME to oppress tumor proliferation and facilitate infiltration and functions of immune cells via inhibiting glycolysis.^204^ Other compounds and drugs include dichloroacetate, indoleamine 2,3‐dioxygenase inhibitors, adenosine receptor antagonists, CD73 inhibitors, etc. These are all directed at distinct facets of tumor cell metabolism to optimize anti‐tumor responses.^205^

#### 4.1.3 Regulation of mitochondrial function and oxidation prevention

*Regulation of mitochondrial function*: Interestingly, lifespan in model organisms could be extended by partially impairing mitochondrial function early in development. A recent study has demonstrated that inhibiting mitochondrial protein synthesis or import via mitochondrial UPR fraction extends lifespan in *C. elegans*.^206^ What's more, inhibitors of the mitochondrial electron transport chain, for instance, metformin could extend *C. elegans* lifespan through mitochondrial hormesis involving peroxiredoxin peroxiredoxin‐2.^163^ Besides, mitophagy renews mitochondria pool and hinders accumulation of injured ones. Mitophagy inducer, for example, urolithin A has been proven to boost insulin sensitivity in mice and extend lifespan in *C. elegans* and aged mice.^164^, ^165^ A first‐in‐human clinical trial showed that UroA injection modified skeletal muscle mitochondrial gene expression and regulated plasma acylcarnitines in sedentary healthy individuals over 4 weeks.^207^ Furthermore, exogenous NAD^+^ treatment increases mitochondrial, cytosolic, and nuclear NAD^+^ levels, while nicotinamide riboside administration specifically raises mitochondrial NAD^+^ levels.^158^

*Oxidation prevention*: Chronic inflammation, accumulation of defective mitochondria and heavy metal, decreased endogenous antioxidants, lower mitophagy efficiency, and proteasomal degradation jointly increase oxidized macromolecules with immunosenescence. Thereby, free radical scavengers and antioxidants, both endogenous (e.g., superoxide dismutase) or exogenous are beneficial.^208^ In a recent study, higher intake of fruits, vegetables, and antioxidants (such as vitamin C, carotenoids, and α‐tocopherol) was found to be linked to lower risk of cardiovascular disease, cancer, and mortality.^171^ However, these foods contain various active substances beyond antioxidants, and benefits are rarely seen in trials with pharmacological antioxidants.^209^ Similarly, ROS could cause lipid peroxidation, especially in polyunsaturated fatty acids.^210^ Deuterated supplementation indeed diminished oxidative stress and lengthened lifespan of *C. elegans*.^211^

#### 4.1.4 Inhibit dysregulated signaling pathways and modulation of proteostasis

*Inhibit dysregulated signaling pathways*: Combining different inhibitors targeting overlapping signaling pathways may be promising. Rapamycin acts as an inhibitor of mTOR1 and prolongs lifespan of model organisms.^166^ Data suggest that rapamycin and its analogs have positive effects on senescence‐related conditions in humans.^79^ Drugs affecting TGF‐β and IGF pathways doubled *C. elegans*’ lifespan.^212^ A combination of MAPK kinase inhibitor trametinib, glycogen synthase kinase‐3 inhibitor lithium, and rapamycin promoted *Drosophila* longevity.^213^ Likewise, short‐term systemic treatment with p38 MAPK inhibitor losmapimod could block monocyte‐derived cyclooxygenase‐2‐driven inflammation, weaken downstream inflammatory processes, and accelerate inflammatory remission.^168^ Recently, senostatics offer a promising method to prevent damage from accumulated senescent cells by modulating their pro‐inflammatory secretions. Initial therapeutic targets include nuclear factor kappa B (NF‐κB), p38, GATA4, mTOR, BRD4, and cGAS/STING, but more specific senostatics are still required.^214^

*Modulation of proteostasis*: The endosome–autophagosom–lysosome pathway is primary protein clearance system for cellular and organismal homeostasis, and therefore, stimulating autophagy is an effective method to eliminate these intracellular aggregates.^215^, ^216^ Several life‐prolonging compounds work as autophagy inducers, including VPS34 PI3‐kinase inhibitors (e.g., wortmannin), histone deacetylase inhibitors (e.g., trichostatin A), MAPK inhibitors (e.g., suberin), TORC1 inhibitors (e.g., rapamycin, everolimus), and adenosine 5′‐monophosphate‐activated protein kinase activators (e.g., metformin, resveratrol).^217^, ^218^ Notably, spermidine showed significant neuro‐ and cardioprotective effects in aging models and humans, suggesting its potential for future human applications.^219^ In addition, a high‐throughput chemical genetics screen of 2750 compounds using a proteasome activity probe identified over ten compounds that boost proteasome activity, among which p38 MAPK inhibitor PD169316 was notably potent.^169^ Various genetic, nutritional, and pharmacological pro‐longevity interventions could regulate adult ECM remodeling and delay senescence‐related decline in collagen expression, generating whole‐body advantages.^220^

#### 4.1.5 Gene therapy and stem cell therapy

*Gene therapy*: The use of gene therapy to alter senescence‐associated genes to promote longevity and a healthy lifespan, with potential clinical applications. Specifically, Bär et al. investigated therapeutic potential of adeno‐associated virus expressing *TERT* in two independent mouse models of aplastic anemia because of telomere shortening.^188^ Another gene target is NF‐κB, which plays a key role in inflammaging and SASP. A study indicated that delivering a lentivirus expressing dominant‐negative IκBα, an NF‐κB inhibitor, into hypothalamus increased mouse lifespan by about 10%.^184^ Furthermore, since senescence is a complex, multi‐stage process involving numerous internal and external factors, combinatorial gene therapy, may enhance protection against immunosenescence. To illustrate, researchers have developed a gene therapy using adeno‐associated virus vectors to co‐express fibroblast growth factor 21, TGF‐β receptor 2, and αKlotho, which has shown synergistic benefits in addressing senescence‐related organ issues such as hypertrophic cardiomyopathy, immune recruitment abnormalities, and disorganized ECM in high‐fat diet‐fed adult mice.^189^ Recently, Koblan et al. utilized in vivo base editing to correct *LMNA* gene pathogenic mutations in cultured fibroblasts derived from children with progeria and in a mouse model of Hutchinson–Gilford progeria syndrome.^190^ Overall, as for in vivo gene therapy, the paramount concern is to determine targets.

*Stem cell therapy*: The body's ability to repair and regenerate tissues declined in stem cell function and reservoir during immunosenescence. Accordingly, transplantation of healthy stem cells into older organisms could antagonize senescence and restore balance in aged organs. Because unfavorable microenvironment in aged organs tends to hinder stem cell maintenance, genetically enhanced stem cells have been designed for transplantation to furnish better regeneration and stress resistance. Namely, transplanting genetically engineered hypothalamic stem cells, expressing dominant‐negative IκBα, has been verified to increase lifespan of 18‐month‐old mice by 10%.^185^ Ensuring safety and effectiveness of these cells is crucial for their use in translational medicine. Thus, FOXO3, a longevity‐linked transcription factor, has been chosen for stem cell engineering therapy. Yan et al. found out that human embryonic stem cell‐derived vascular cells could be functionally enhanced by engineering them to express an activated form of FOXO3, exhibiting delayed senescence, greater oxidative stress, and tumor transformation resistance.^186^

#### 4.1.6 Nutritional and microbiome interventions

*Nutritional interventions*: There are interactions between nutrition, immune response, and inflammatory condition, which are closely related to the characteristics of immunosenescence.^48^ Older individuals often experience nutritional deficiencies, consequently placing them at an increased risk of immunosenescence. Interestingly, dietary additions such as vitamins, micronutrients, and omega‐3 fatty acids have been unraveled as paramount in innate and adaptive immunity.^221^ Mediterranean diet, as an immunosenescence‐delaying diet, sustained intake of which encourages longevity, affects immune homeostasis preservation, and bolsters directly to the decline of inflammation and metabolic disorders.^177^ According to research different forms of dietary restriction such as calorie restriction seem to be quite rosy modulators of immune system.^222^ Calorie restriction diets could down‐regulate pro‐inflammatory molecules related to inflammaging and shortened lifespan, including IL‐6, TNF‐α, and IL‐1β.^178^ Similarly, studies have attested that replenishment with prebiotics or probiotics accelerates antibody responses to vaccination and produces innate immune responses to infection.^48^ To identify nutritional tactics that both favor immunity and minimize side effects in old characters. Numerous studies are currently underway to examine the role of multiple nutritional interventions in modifying immune system characteristics and functions in older folks. Research shows that omega‐3 polyunsaturated fatty acids could lengthen leucocyte telomeres in the elderly, reducing multifold immunosenescence markers and further decreasing systemic inflammaging.^172^, ^173^ Nutrients interact uniquely with GALT in the intestines. Enterocytes in the intestinal barrier could detect antigens from nutrients and microbiota, passing them to the immune system in lamina propria, which then initiates immune responses. Nutrients influence innate immunity and inflammation by regulating TLRs and cytokines, affecting immune cell crosstalk and signaling. They also impact adaptive immunity by modulating B and T‐cell differentiation, proliferation, activation, and antibody production.^223^

*Microbiome interventions*: The gut's commensal microflora significantly influences immune homeostasis.^224^ Thus, using probiotics, prebiotics, and their combination (symbiotics) to modulate immune system by affecting microbiota is common. Castro‐Herrera et al. found that elderly subjects had better responses to seasonal influenza vaccination after a year of probiotic therapy (e.g., *Lacticaseibacillus* and *Bifidobacterium*).^225^ Additionally, giving healthy elderly individuals *Lactobacilli* and soluble corn fiber boosted NK cell responses and reduced systemic inflammaging.^174^ Studies on pro‐ and prebiotic supplementation in older people indicate reduced inflammation, with lower TNF‐α, IL‐1β, and IL‐6 levels and higher IL‐10 levels. These pro‐ and prebiotics also enhance innate immune responses, improving phagocytosis, cytotoxicity against bacteria such as *Staphylococcus aureus*, increasing NK cell activity, and reducing CD25 expression in resting T lymphocytes.^226^

#### 4.1.7 Exercise and physical activity

Until very recently, it offered a renewed direction that regular physical exercise could decrease blood inflammatory cytokines levels and augment innate immune responses, decrease exhausted T‐cell number, and increase T‐cell proliferation in the elderly.^179^ Aggrandizing thymic mass and naïve T‐cell output by stirring IL‐7 and growth hormone synthesis could be a hidden mechanism by which exercise governs immunosenescence hallmarks. Furthermore, muscle is an instrumental regulator of immune system. IL‐15 produced by muscle accelerates cytotoxicity and cytokine secretion by NK cells and sustains T and NK cell numbers in blood. There is also a skeletal muscle that generates a protein termed myokines that has anti‐inflammatory and immune response‐boosting properties. By employing a similar strategy, Shen et al. unveiled that osteoblast and lymphocyte niches exhausted due to immunosenescence around the bone marrow could be arrested through the mechanical load of exercise.^227^ Exercise could mobilize and redistribute effector T cells relying on a combination of catecholamines with β‐2 adrenergic receptors to replenish immune responses.^228^ Older persons are encouraged to facilitate more exercise as far as their capabilities.^229^

### 4.2 Counteract immunosenescence to reverse functions of immune cells

#### 4.2.1 Innate immune cells

*Neutrophils*: Several studies have proven the possibility of restoring immunosenescence‐induced neutrophil disequilibrium. Pharmacological interventions such as statins which are directed at cell division control protein 42 (CDC42) have been explored to amend neutrophil chemotaxis and diminish neutrophil extracellular trap lesions.^170^ Besides, regulation of nutrition intake such as vitamin E has been ensured to improve chemotaxis, while eicosapentaenoic acid intake appears to reduce ROS bursts in the aged.^175^ Permanent physical exercise also enhances neutrophil functions by gaining resistance to immunosenescence.^48^

*Monocytes/macrophages*: p38 MAPK signaling pathway inhibitors or other cyclooxygenase‐2 inhibitors seem to revive immune response of monocytes.^230^ Pharmacological options such as flavonoid‐rich cocoa polyphenols have been found to reduce expression of adhesion molecules and pro‐inflammatory markers on monocytes.^170^ Long‐term exercise could reduce the production of inflammatory monocyte subpopulations and pro‐inflammatory cytokines, thereby counteracting immunosenescence.^48^ In terms of macrophages, repolarization from M2 to M1 could generate pro‐inflammatory implications to clear immunosenescent cells, appearing more profound in counteracting immunosenescence.^231^ More recently, it has been demonstrated that IL‐4 therapy could prevent macrophage senescence and improve healthy lifespan in aged mice, while type 2 cytokine signaling likewise has curative potency to amend healthy aging.^232^

*APCs*: The employment of TLR agonists as adjuvants appears to be a prospective tactic for DCs against immunosenescence. TLR agonists have been proven to facilitate T‐cell activation by boosting antigen uptake, presentation, and DCs’ maturation, as well as cytokine secretion.^233^ To date, quite a few TLR agonists are already administered as adjuvants in licensed vaccines, and others are under development.

*NK cells*: NK cells are instrumental in filling gap between innate and adaptive immunity, and diverse studies have revealed an essential function for NK cells in eliminating senescent tumor cells.^234^ NK group 2, member D (NKG2D), a receptor has been intensively studied in these processes, and use of certain immunosenescence‐inducing chemotherapeutic agents could reward the excessive expression of these NKG2D ligands in a wide range of tumor types.^235^ Likewise, physical activity and dietary interventions are candidates to minimize NK cell immunosenescence. These factors increase NK cell frequency and enhance NK cell differentiation and NK cytotoxic activity. Additionally, zinc supplementation could recover NK lysis activity, and supplementation with vitamins B9 and B12 could scale up the number and activity of NK cells.^176^

#### 4.2.2 Adaptive immune cells

*B cells*: One plausible strategy for B cells to counteract immunosenescence is to enhance autophagy. Immunosenescent B lymphocytes could induct decreased levels of autophagy and obstructed B‐cell responses, but treatment with spermidine could restore their functions instead.^236^ Inspiringly, B‐cell subpopulations, repertoire, cellular functions in vitro, and immune responses in vivo were analyzed using depletion of blood B cells in older and younger mice models. Results found that generating rejuvenated B cells in bone marrow could cover cyto‐responsiveness to immune stimuli in vitro.^237^

*T cells*: An underlying immunotherapeutic tactic could target altered molecules to reverse T‐cell senescence. The most obvious target for T‐cell senescence reversal is p38 MAPK, given that CD4^+^ and CD8^+^ T cells are at the heart senescence signaling.^238^ Targeting the p38 MAPK pathway could also restore TCR functions and revert CD8^+^ T‐cell immunosenescence through a mTOR‐independent process.^167^ Others also target changed molecules in immunosenescent T cells, genetically inhibiting dual‐specificity phosphatase (DUSP) 6 or DUSP4 could revive T‐cell signaling, etc.^239^

Another latent strategy is to reshape T‐cell immunosenescence by targeting cell signaling. Encouragingly, suppressing mTOR upstream activator VPS39 could increase level of memory T cells and foster antigen‐specific T‐cell amplification.^240^ It has been elucidated that drug interventions, for example, using drugs that favor lipolysis or blocking phospholipase A2, and cytosolic to remodel lipid metabolism may prevent T‐cell decline.^241^ Exercise and nutrients respectively have been proven to reduce immunosenescent lymphocytes and improve the functions and responses to vaccination.^242^, ^243^ Moreover, adoptive T‐cell therapy which amplifies and translocates stem cells, memory cells, or virus‐specific T cells in vitro to older adults has also been considered as an underlying strategy to confront T‐cell immunosenescence.^244^

### 4.3 Counteract immunosenescence to reverse functions of immune organs

#### 4.3.1 Central immune organs

*Thymus*: Reduction of Forkhead box N1 (FOXN1), an indispensable transcription factor for thymocyte differentiation and progression, could cause thymic regression, and the transcription factor is adversely connected with age. It could decrease thymic deterioration through gene therapy or transplantation of thymocytes with a high FOXN1 level and restore thymic activity by indirectly augmenting the number of immature T cells simultaneously. Equivalently, FOXN1 has been applied as a pivotal target for reversing thymic senescence.^245^ Moreover, a preclinical assay in mouse models underlined efficacy in recovering thymic functions and amplifying peripheral immune cells after immune injury.^246^

*Bone marrow*: Studies were conducted on the shifts in phenotype and functions of adaptive immune system and large amounts of ROS fueled by HSCs senescence.^247^ CDC42 has been illuminated to be a dominating factor in HSC senescence. A confirmed potent ROS inducer is p38 kinase, which could be inhibited to restore HSC potency. Consequently, suppressing CDC42 and targeting p38 kinase is the principal avenue to reverse HSC senescence.^248^ Pleasantly, Kuribayashi et al. harbored the idea that transplantation of older HSCs into younger niches reinstated the immunosenescent transcriptional profile of HSCs.^249^ Moreover, the repopulation of bone marrow with autologous induced pluripotent stem cell‐derived HSCs is a potentially promising method to revitalize a large part of immune system, particularly innate immune response.^250^

#### 4.3.2 Peripheral immune organs

*LNs*: Despite some studies to restore B‐cell compartment vigor, it remains overwhelmingly challenging to recover immunocompetence and vaccination responses in immunosenescent models. It has been hypothesized that NK cell immunity is largely restored by killer cell lectin‐like receptor C1 (NKG2A) inhibition. In addition, recombinant IL‐7 was manifested in clinical trials to recover a physiological level in T cells.^251^ Anti‐fibrotic or senolytic drugs may reduce LN fibrosis and reshape its degenerated structure. Furthermore, synthetic LNs could robustly capture the features in vivo to regain immune functions.^252^ This method stands a chance of opening renewed potentials which could be coupled with other tactics for enduring immune function rejuvenation.

*Spleen*: Yousefzadeh et al. discovered that transplanting splenocytes from young mice reduces senescence and tissue damage in older mice.^253^ What's more, SIRPα DCs have been revealed to regulate homeostasis of fibroblastic reticular cells via TNF receptor ligands in white pulp of adult spleen.^254^ However, there is still a paucity of pertinent studies, and understanding the molecular causes of immunosenescence‐related changes in the spleen and LNs could help develop new treatments.

*MALTs*: In the aged, tremendous immunosenescent alterations, namely, decreased barrier functions, epithelial cell proliferation, stem cell retention, increased intestinal leakiness, and pro‐inflammatory dysbiosis take place in the gut, leading to further exacerbated inflammaging and pro‐inflammatory states.^255^, ^256^ There are some approaches to combat or pre‐empt the deterioration of microbiota, for instance, supplementing prebiotics which could boost beneficial microbiota and decrease immunosenescent microbiota.^257^ A secretory IgA antibody elicited in upper respiratory pathway together with mucosal delivery could be another approach for MALTs to compete against immunosenescence through the neutralization of the virus and cross‐protection of mutant strains.^258^

## 5 NEW ADVANCES IN IMMUNOSENESCENCE FOR TUMOR IMMUNOTHERAPY

Immunotherapies targeting TIME have shifted immunologic landscape of cancer treatment. Current treatments target host's immune system, mobilizing immune cells against cancer. The distinguishing feature of immunotherapy is its long‐lasting efficacy, which may be because of adaptive immune memory, thus enabling some patients to survive for long periods.^259^ As immune profiles and functions alter significantly with immunosenescence, targeting immune system in the elderly has important clinical implications.^1^ Table 3 summarizes current clinical trial landscape exploring new advances in immunosenescence for tumor immunotherapy.

**TABLE 3:** Current clinical trial landscape exploring new advances in immunosenescence for tumor immunotherapy.

NCT number	Study title	Study status	Conditions	Interventions	Phases	Study results
(A) Immune checkpoint blockade						
NCT06410534	A phase II study evaluating an organ preservation strategy using immune checkpoint blockade for participants with primary colorectal or gastroesophageal cancer	NOT_YET_RECRUITING	Colorectal cancer Gastroesophageal cancer	DRUG: nivolumab DRUG: ipilimumab	II	–
NCT05879484	Study of front line pembrolizumab and valemetostat in PD‐L1 positive, HPV‐negative recurrent/metastatic SCC of the head and neck: the PANTHERAS	NOT_YET_RECRUITING	Sinonasal cancer Squamous NSCLC Lung cancer Head and neck SCC Head and neck carcinoma Head and neck cancer	DRUG: pembrolizumab DRUG: valemetostat	I/II	–
NCT05589818	Pembrolizumab for advanced NSCLC and PS 2−3	RECRUITING	NSCLC	DRUG: pembrolizumab	II	–
NCT03673332	Elderly cancer patients, safety and quality of life under immunotherapies	UNKNOWN	Advanced or metastatic melanoma Advanced or metastatic NSCLC	DRUG: immune‐checkpoint inhibitors therapies	IV	–
NCT03416244	A multicenter open‐label phase II trial to evaluate nivolumab and ipilimumab for second‐line therapy in elderly patients with advanced esophageal squamous cell cancer	COMPLETED	Esophageal cancer Oesophageal cancer Oesophageal cancer metastatic Esophageal cancer metastatic Esophageal cancers NOS Oesophageal cancer NOS Gastroesophageal cancer Gastrooesophageal cancer	DRUG: nivolumab DRUG: ipilimumab	II	–
NCT03293680	Pembrolizumab in elderly patients with advanced lung cancer	ACTIVE_NOT_RECRUITING	NSCLC	DRUG: pembrolizumab	II	–
NCT02374242	Anti‐PD‐1 brain collaboration for patients with melanoma brain metastases	ACTIVE_NOT_RECRUITING	Melanoma Brain metastases	DRUG: nivolumab DRUG: ipilimumab	II	–
(B) Adoptive cell therapies						
NCT06364423	Anti‐CD19 chimeric antigen receptor T‐cell immunotherapy for CLL	NOT_YET_RECRUITING	B‐cell CLL Leukemia, lymphocytic, chronic, B‐cell B‐lymphocytic leukemia, chronic	BIOLOGICAL: autologous HuCD19 (anti‐CD19) CAR T cells DRUG: cyclophosphamide DRUG: fludarabine DRUG: rituximab	I/II	–
NCT06204991	To evaluate the safety and efficacy of ADP‐TILIL7 in patients with locally advanced or metastatic melanoma	NOT_YET_RECRUITING	Melanoma stage III Melanoma stage IV Melanoma	BIOLOGICAL: ADP‐TILIL7 DRUG: cyclophosphamide DRUG: fludarabine phosphate DRUG: proleukin	I	–
NCT05797233	Trial of anti‐CD19 and anti‐CD20 bicistronic chimeric antigen receptor T cells for treating B‐cell malignancies	RECRUITING	CLL B‐cell CLL Lymphoma, B cell	BIOLOGICAL: anti‐CD19 and anti‐CD20 bicistronic CAR T cells DRUG: cyclophosphamide DRUG: fludarabine	I	–
NCT05003895	GPC3 targeted CAR‐T cell therapy in advanced GPC3 expressing hepatocellular carcinoma	RECRUITING	Hepatocellular carcinoma Hepatocellular cancer Metastatic hepatocellular carcinoma	DRUG: cyclophosphamide BIOLOGICAL: CAR‐T cell DRUG: fludarabine	I	–
NCT04537442	Clinical study to evaluate the safety and efficacy of IM21 CAR‐T cells in the treatment of elderly patients with relapsed or refractory multiple myeloma	UNKNOWN	Multiple myeloma	DRUG: IM21 CAR‐T cells	I	–
NCT04209829	Response to CAR‐T cells therapy in patients with hematologic malignancies depending on tumor characteristics	NOT_YET_RECRUITING	Hematologic diseases			–
NCT02799550	Allogeneic CART‐19 for elderly relapsed/refractory CD19^+^ ALL	UNKNOWN	Leukemia	BIOLOGICAL: allogeneic CART‐19	I	–
(C) Bi‐specific T‐cell engagers						
NCT03476239	Efficacy and safety of the BiTE antibody blinatumomab in Chinese adult subjects with relapsed/refractory B‐precursor acute lymphoblastic leukemia	COMPLETED	ALL	DRUG: blinatumomab DRUG: dexamthasone	III	YES
NCT01207388	Confirmatory phase II study of blinatumomab (MT103) in patients with minimal residual disease of B‐precursor acute lymphoblastic leukemia	COMPLETED	B‐cell ALL	DRUG: blinatumomab	II	YES
NCT00560794	Phase II study of the BiTE blinatumomab (MT103) in patients with minimal residual disease of B‐precursor acute lymphoblastic leukemia	COMPLETED	ALL	BIOLOGICAL: blinatumomab (MT103)	II	YES
NCT00274742	Safety study of the bispecific T‐cell engager blinatumomab (MT103) in patients with relapsed non‐Hodgkin's lymphoma	COMPLETED	Non‐Hodgkin's lymphoma, relapsed	BIOLOGICAL: blinatumomab (MT103)	I	YES
NCT06359067	A real‐world study of bispecific antibodies in multiple myeloma	COMPLETED	Multiple myeloma	OTHER: treated by bispecific antibodies, teclistamab or elranatamab in usual care OTHER: not treated by bispecific antibodies in usual care		–
NCT06062537	Analysis of effectiveness and safety of teclistamab in relapsed and refractory multiple myeloma patients	RECRUITING	Multiple myeloma			–
NCT03145181	Dose escalation study of teclistamab, a humanized BCMA*CD3 bispecific antibody, in participants with relapsed or refractory multiple myeloma	RECRUITING	Hematological malignancies	DRUG: teclistamab (IV) DRUG: teclistamab (SC)	I	–
NCT05315258	Tebentafusp in molecular relapsed disease melanoma	RECRUITING	Melanoma (skin) Melanoma, uveal	DRUG: tebentafusp	II	–
(D) High‐dose cancer vaccines						
NCT00003568	Vaccine therapy with high‐dose IL‐2 in treating patients with metastatic melanoma	COMPLETED	Melanoma (skin)	BIOLOGICAL: aldesleukin BIOLOGICAL: gp100 antigen BIOLOGICAL: incomplete Freund's adjuvant	II	–
(E) Adjuvanted cancer vaccines						
NCT06252584	Multi‐peptide vaccination adjuvanted with XS15 in acute myeloid leukemia patients	NOT_YET_RECRUITING	Acute myeloid leukemia, adult	DRUG: vaccines, peptide	I	–
NCT03771157	Serologic response to SHINGRIX vaccine in patients with CLL and WM treated with BTK inhibitors	COMPLETED	CLL WM	DRUG: Shingrix vaccine	I	YES
NCT03633110	Safety, tolerability, immunogenicity, and antitumor activity of GEN‐009 adjuvanted vaccine	COMPLETED	Cutaneous melanoma NSCLC SCC of the head and neck Urothelial carcinoma Renal cell carcinoma	BIOLOGICAL: GEN‐009 adjuvanted vaccine DRUG: nivolumab DRUG: pembrolizumab	I/II	–
(F) Vector‐based cancer vaccines						
NCT06319963	A study to evaluate lenti‐HPV‐07 immunotherapy against HPV^+^ cervical or oropharyngeal cancer	NOT_YET_RECRUITING	HPV‐related cervical carcinoma HPV‐positive oropharyngeal SCC	DRUG: two IM injections Lenti‐HPV‐07 DRUG: one IM injection Lenti‐HPV‐07	I/II	–
NCT05553639	HB‐302/HB‐301 therapy in participants with metastatic castration‐resistant prostate cancer	RECRUITING	Prostate cancer metastatic	BIOLOGICAL: HB‐302/HB‐301 alternating 2‐vector therapy	I/II	–
NCT05108870	TheraT vectors (vaccines) combined with chemotherapy to treat HPV16 head and neck cancers	RECRUITING	HPV HPV‐positive oropharyngeal SCC Head and neck cancer	DRUG: HB‐201 DRUG: HB‐202 DRUG: carboplatin DRUG: paclitaxel PROCEDURE: transoral robotic surgery	I/II	–
NCT03141463	Vvax001 cancer vaccine in (pre) malignant cervical lesions	COMPLETED	CIN 2/3 Cervical cancer	BIOLOGICAL: Vvax001 therapeutic cancer vaccine	I	–
NCT01856920	QUILT‐3.006 for recurrent medullary thyroid cancer	UNKNOWN	Medullary thyroid cancer	BIOLOGICAL: GI‐6207 (recombinant *Saccharomyces cerevisiae*‐CEA [610D])	II	–
(G) mRNA cancer vaccines						
NCT06141369	Treatment of advanced endocrine tumor with individualized mRNA neoantigen vaccine (mRNA‐0523‐L001)	RECRUITING	Adrenal cortical carcinoma Medullary thyroid cancer Thymic neuroendocrine carcinoma Pancreatic neuroendocrine tumor	BIOLOGICAL: individualized mRNA neoantigen vaccine (mRNA‐0523‐L001)	–	–
NCT05198752	A study of neoantigen mRNA personalized cancer in patients with advanced solid tumors	RECRUITING	Solid tumor	DRUG: neoantigen mRNA personalized cancer SW1115C3	I	–
NCT03908671	Clinical study of personalized mRNA vaccine encoding neoantigen in patients with advanced esophageal cancer and NSCLC	RECRUITING	Esophageal cancer NSCLC	BIOLOGICAL: personalized mRNA tumor vaccine	–	–
NCT03480152	mRNA‐based, personalized cancer vaccine against neoantigens expressed by the autologous cancer	TERMINATED	Melanoma Colon cancer Gastrointestinal cancer Genitourinary cancer Hepatocellular cancer	BIOLOGICAL: National Cancer Institute‐4650, a mRNA‐based, personalized cancer vaccine	I/II	YES
NCT01278940	Trial of vaccine therapy with mRNA‐transfected dendritic cells in patients with advanced malignant melanoma	COMPLETED	Malignant melanoma	BIOLOGICAL: dendritic cells malignant melanoma PROCEDURE: IL‐2	I/II	–
(H) Personalized cancer vaccines						
NCT03794128	A study of personalized neoantigen cancer vaccines	COMPLETED	NSCLC Colorectal cancer Gastroesophageal adenocarcinoma Urothelial carcinoma Pancreatic ductal adenocarcinoma	PROCEDURE: blood collection for research (next‐generation sequencing) PROCEDURE: blood collection for research (HLA typing)	–	–
NCT03631043	Personalized vaccine in treating patients with smoldering multiple myeloma	ACTIVE_NOT_RECRUITING	Smoldering plasma cell myeloma	PROCEDURE: biopsy specimen radiography DRUG: lenalidomide BIOLOGICAL: vaccine therapy	I	–
NCT03558945	Clinical trial on personalized neoantigen vaccine for pancreatic tumor	RECRUITING	Pancreatic tumor	BIOLOGICAL: personalized neoantigen vaccine	I	–
NCT02808416	Personalized cellular vaccine for brain metastases (PERCELLVAC3)	COMPLETED	Brain cancer Neoplasm metastases	BIOLOGICAL: personalized cellular vaccine	I	–
NCT02709616	Personalized cellular vaccine for glioblastoma (PERCELLVAC)	COMPLETED	Glioblastoma	BIOLOGICAL: personalized cellular vaccine	I	–

*Note*: Symbol “‒” denotes no results available.

Abbreviations: 6MHP, a vaccine containing 6 melanoma‐associated peptides to stimulate helper T cells; ALL, acute lymphocytic leukemia; BiTE, bi‐specific T‐cell engager; BTK, bruton's tyrosine kinase; CAR, chimeric antigen receptor; CIN, cervical intraepithelial neoplasia; CLL, chronic lymphocytic leukemia; HLA, human leukocyte antigen; HPV, human papilloma virus; IL, interleukin; IM, intramuscular; IV, intravenous; NCT, National Clinical Trials; NOS, Newcastle‐Ottawa Scale; NSCLC, non‐small cell lung cancer; PD‐1, programmed cell death protein‐1; PD‐L1, programmed cell death 1 ligand‐1; PS, performance status; SC, subcutaneous; SCC, squamous cell carcinoma; WM, Waldenstrom macroglobulinemia.

*Source*: https://clinicaltrials.gov/.

### 5.1 IMMUNOSUPPRESSION‐REDUCING TREATMENTS

Promising therapeutic targets appear to be immunosuppressive cells, particularly MDSCs and Tregs to enhance effectiveness of immunotherapy.^260^, ^261^ For MDSCs, these strategies include normalizing production, promoting maturation, reducing trafficking and expansion, inhibiting suppressive activities, and depleting MDSCs number in tumors. Salminen et al. summarized MDSC‐dependent anti‐tumor and anti‐inflammatory properties of various phytochemicals, comprising flavonoids, terpenoids, retinoids, curcumins, and β‐glucans.^262^ These phytochemicals targeting signal transducer and activator of transcription 3 and NF‐κB signaling inhibit functions of MDSCs. Encouragingly, retinoic acid and β‐glucans could promote MDSCs maturation into non‐immunosuppressive macrophages and DCs.^263^ Moreover, signal transducer and activator of transcription 3 inhibitors such as sunitinib and axitinib have reduced MDSC accumulation in tumors and enhanced anti‐tumor effects.^264^

Several strategies could inhibit Treg functions, including: (1) targeting immune checkpoint receptors. Research has demonstrated that TME enhances suppressive function of Treg cells by increasing expression of immune checkpoint molecules (e.g., programmed cell death protein‐1 [PD‐1]), making these molecules viable targets for cancer treatment.^265^ (2) Shifting to anti‐tumor T‐cell phenotypes. Kachler et al. revealed that TGF‐β converts IFN‐γ‐producing Tbet^+^Th1 CD4^+^ T cells into immunosuppressive Tbet and Foxp3‐PD‐1 co‐expressing regulatory cells, thereby blocking anti‐tumor immune functions in both mice and humans. Targeting these TbetFoxp3CD4 T cells could enhance current lung cancer immunotherapy.^266^ (3) Targeting specific proteins such as Foxp3. As Foxp3 Tregs inhibit anti‐tumor response in endogenous lymphomas, ablation of Foxp3 Tregs significantly delays tumor progression.^267^ (4) Disrupting metabolism. It was experimentally evidenced that 3‐oxoLCA and isoalloLCA administration to mice diminished Th17 cell differentiation and augmented Treg cell differentiation in intestinal lamina propria.^268^ What's more, chemotherapeutic agents with leukocyte‐depleting effects such as cyclophosphamide or low‐dose temozolomide could improve effectiveness of anti‐tumor CTL by lessening number of Tregs.^269^

### 5.2 Immune checkpoint blockade

ICIs such as PD‐1 and its ligand‐1 (PD‐L1) inhibitors and CTL antigen‐4 (CTLA‐4) have been validated to elicit potent anti‐tumor activity as well as outcomes in various cancers. PD‐1 and CTLA‐4 receptors could inhibit effector T cells when activated. The expression of immune checkpoint molecules such as PD‐1, Lag3, and Tim3 on exhausted T cells increases with immunosenescence.^270^, ^271^ Additionally, PD‐L1 expression in CD8 effector T cells is significantly elevated in aged mice compared to young mice.^272^ Anti‐PD‐L1 treatment was shown to enhance in vitro proliferation and anti‐tumor immunity in aged hosts more effectively than in young hosts within a mouse lymphoma model.^273^

A thorough review of data shows that currently accessible ICIs are remarkably potent in the elderly.^274^, ^275^ A study conducted by Kugel et al. found that melanoma patients over 60 years had a better response to anti‐PD‐1 therapy, with response likelihood increasing with immunosenescence, this was also observed in both young and aged mouse models bearing YUMM melanoma.^272^ Immunotherapeutic responses may differ significantly between younger and older patients depending on diverse immune cell profiles and tumor environments. Nevertheless, Rarely have studies directly examined these differences. Additionally, varying effectiveness of targeting different ICIs in aged models suggests that age‐specific immunotherapy may be justified.^276^ Inspiringly, senescent tumor cells that avoid NK cell immunity by shedding NKG2D ligands or increasing HLA‐E molecules could be targeted with developing antibodies that block NKG2A checkpoint or prevent NKG2D ligand MICA/B shedding.^200^, ^277^, ^278^, ^279^

### 5.3 ACT

ACT involves infusing ex vivo‐expanded, autologous, or allogeneic immune cells into patients to eliminate tumors. Common ACT types include tumor‐infiltrating lymphocytes and gene‐modified T cells, like those with transgenic TCRs or chimeric antigen receptors (CARs). Unlike other ACT types, CAR‐T cells are not major histocompatibility complex (MHC) dependent and hence could be engineered to target any tumor‐associated antigen. Apart from stem cell therapy mentioned aforesaid to replenish or rejuvenate damaged stem cells, an alternative senescence‐protective strategy is to eliminate abnormal senescent cells, as they generate an inflammatory environment resulting in organ defects. Identifying surfaceome in senescent cells has revealed potential targets such as urokinase plasminogen activator receptor (uPAR),^280^ DPP4,^281^ NOTCH1,^282^ membrane‐bound Vimentin,^283^ etc.

Amor et al. found that uPAR is a cell surface protein widely induced during immunosenescence. Accordingly, uPAR‐specific CAR‐T cells could effectively eliminate these cells both in vitro and in vivo. These CAR‐T cells raised survival in mice with lung adenocarcinoma receiving senescence‐inducing drugs and recovered tissue health chemical‐ or diet‐induced liver fibrosis.^280^ This highlights therapeutic aptitude toward immunosenescence by senolytic CAR‐T cells.^284^ Besides, induced pluripotent stem cell‐derived cells engineered with CARs also show promise for next‐generation cellular immunotherapies.^285^ A CAR‐T product from induced pluripotent stem cells is now in a clinical trial for B‐cell malignancies.^286^

Landmark trials and real‐world evidence show that CAR‐T therapy maintains high response rates in patients aged 65 and older, but may increase toxicity.^287^ It remains to be elucidated what interventions could improve treatment outcomes for older adults treated with CAR‐T. Nevertheless, evidence shows that immunosuppressive microenvironment created by solid tumors could hinder CAR‐T‐cell therapy.^288^ Consequently, combining CAR‐T cells with histone deacetylase inhibitors may be a potential solution, as demonstrated by Ali et al., that could enhance CAR‐T cell anti‐tumor effects by promoting their differentiation into central memory cells in pancreatic cancer of murine models.^289^ To date, a plethora of gene modification strategies have been used to augment therapeutic efficacy of ACT in solid tumors, engineering NK cells or macrophages to express NK cell receptors, transgenic TCRs, CARs, or TCR‐like CARs.

### 5.4 BiTEs

BiTEs are recombinant proteins with two antigen‐binding modules, one targeting a tumor‐specific antigen, and the other targeting a T‐cell activating molecule such as CD3. Dual binding of modules activates T cells and forms immune synapses with tumor cells, leading to tumor cell lysis.^290^ Blinatumomab, which targets CD3 and CD19, received FDA approval in 2014 and EMA approval in 2015 for treating Philadelphia chromosome‐negative B‐cell acute lymphoblastic leukemia.^291^ Inspiringly, it is expected that elderly patients will similarly benefit from this therapy. Recent data indicated that CD80 or CD86 expressed on cancer cells could enhance BiTE cytotoxicity in vitro by signaling through CD28, as does concomitant use of monoclonal CD28 antibodies.^292^ This highlights importance of CD28 activation for optimal anti‐tumor efficacy of BiTEs. Whereas effectiveness of BiTEs in humans may be restricted by loss of CD28 expression on T cells, which occurs during lymphocyte senescence.^293^ Currently, FDA has approved three BiTE molecules: blinatumomab and teclistamab, which target tumor‐associated antigens via antibody‐derived binding domains, and tebentafusp, which targets gp100 peptides in the MHC class I complex using a high‐affinity TCR.^294^, ^295^

### 5.5 Cancer vaccines

Cancer vaccines could generate vigorous tumor‐specific immune responses and serve as a satisfactory therapy due to the specificity for tumor cells and durable immune memory that may safeguard against recurrences.^296^ In this section, we outline current strategies to enhance cancer vaccine efficacy, as vaccination potency is still far from optimal for the older population.

*High‐dose vaccines*: One of the immunodeficiencies with immunosenescence is a remission in immune synapse formation, which produces an implication on attenuated antigen presentation. Higher antigen doses are hypothesized to reinforce antigen presentation by innate immune cells, subsequently improving antibody production as well as adaptive immune responses.^297^ High‐dose vaccines could surmount antigen delivery deficiencies by promoting APCs to B cells, eliciting elevated antigen‐specific antibody responses.^106^

*Adjuvanted vaccines*: Adjuvant is an army of ingredients that augment the vaccine immunogenicity when used in combination with vaccine antigens.^298^ There are other diverse categories of components that have been assessed as adjuvants, namely, mineral salts, microbial products, emulsions, saponins, synthetic small‐molecule agonists, polymers, nanoparticles, and liposomes.^299^ They have been ascertained in pre‐clinical and clinical studies to sustain strength, breadth, together with immune responses’ durability.^300^ Aiming at innate immune cells and mobilizing pattern recognition receptors signaling pathways, adjuvants could direct and augment specific adaptive immune responses.^301^ Specifically, certain delivery agents could alternatively act as adjuvants to spur antigen uptake and presentation by mimicking spatial structure and size of natural pathogens.^302^ Significantly, numerous nanoparticle drug delivery systems could induce the most productive antibody responses by aiming directly at B cells.^303^ Since a sole adjuvant might not be able to supply implications simultaneously, it is often selected to harness the combination of immunostimulatory adjuvants and delivery system adjuvants for formulating cancer vaccines. Another strategy to boost cancer vaccine efficacy is combining them with systemic immunostimulant adjuvants, for example, cytokines, particularly IL‐2 or granulocyte‐macrophage colony‐stimulating factor.^299^, ^304^

*Vector‐based vaccines*: Using viral vector vaccines as an example, non‐pathogenic viruses to genetically codify external transgenes could be designed and represent encoded antigens utilizing host cell apparatus. Viral vector vaccines are highly immunogenic and able to induce antigen‐specific B and T‐cell immunity.^305^ Some viral vectors harbor added advantage of versatility owing to more antigens on structural capsule components of viral surface. Viral vectors have potential to be alternative tools for preventing infectious diseases and tumors since they can be programmed to express or encode single or multiple antigens.

*mRNA vaccines*: mRNA vaccine is a novel oncotherapy combining molecular biology with immunology. After incorporating mRNA into lipid nanoparticles, it is conveyed to the host cells via intramuscular injection.^306^ Intracellularly, mRNA is translated into antigenic proteins and after that processed into peptides either by endosomal cleavage or proteasomal degradation and finally represented by MHC molecules at plasma membrane, resulting in an immune response.^307^ mRNA vaccines possess plentiful structural variations on the basis that mRNA molecules either contain only target antigens (conventional mRNA vaccines) or target antigens are further amplified by mRNA molecules upon entry into host cells with a replicative mechanism (self‐amplifying or trans‐amplifying) for prolonged and abundant antigenic expression.^308^

**FIGURE 5:**
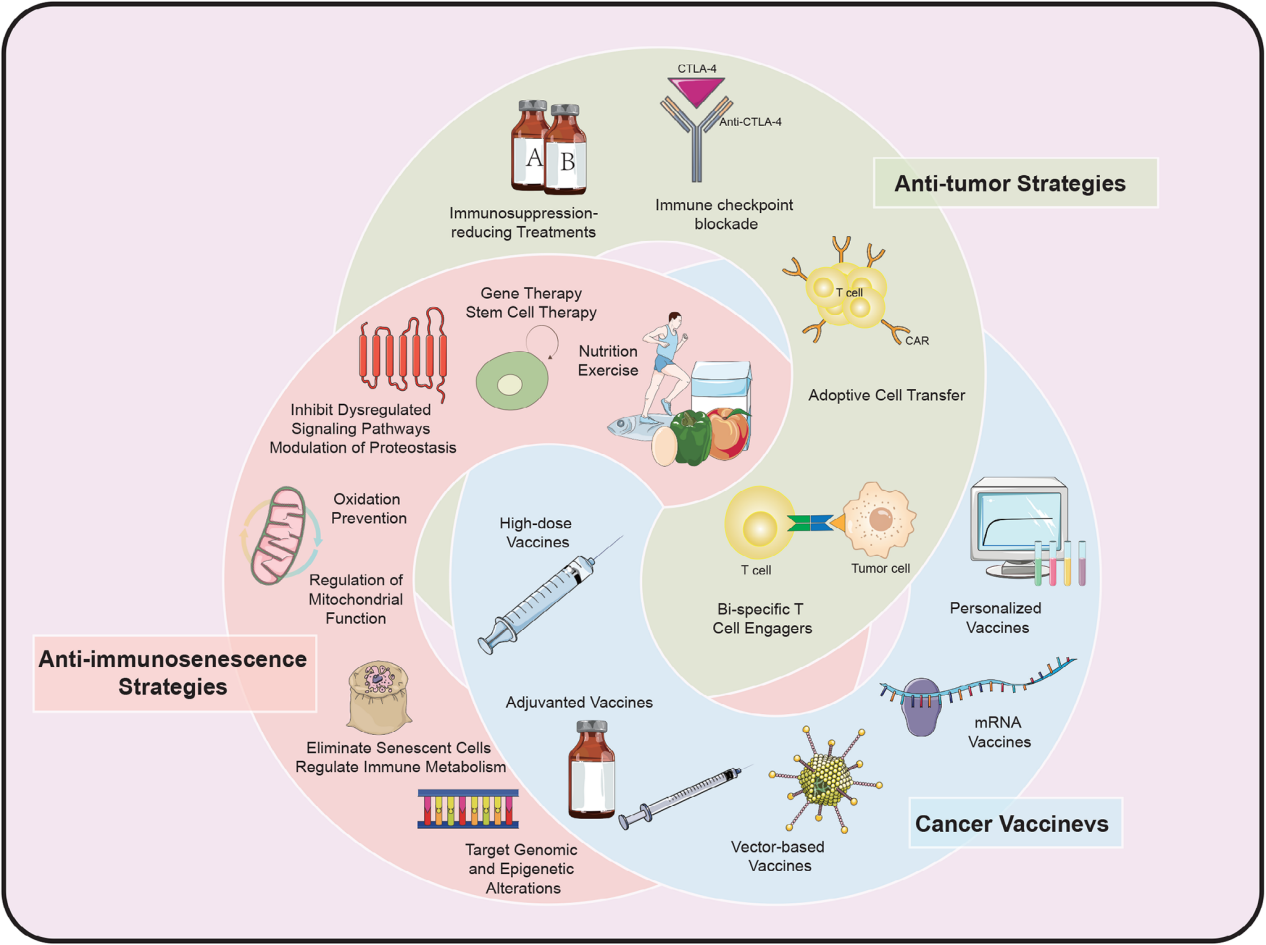
Anti‐immunosenescence and anti‐tumor strategies for the elderly. Anti‐immunosenescence strategies include repression of genomic instability and targeting immune epigenetics, elimination of senescent cells and regulate immune metabolism, regulation of mitochondrial function and oxidation prevention, inhibit dysregulated signaling pathways and modulation of proteostasis, gene therapy and stem cell therapy, nutritional and microbiome interventions, and exercise and physical activity. New advances in immunosenescence for tumor immunotherapy comprise immunosuppression‐reducing treatments, adoptive cell transfer, bi‐specific T‐cell engagers, and cancer vaccines (high‐dose vaccines, adjuvanted vaccines, vector‐based vaccines, mRNA vaccines, personalized vaccines). These strategies are closely interrelated and jointly improve treatment paradigms and quality of life in oncology for older patients.

*Personalized vaccines*: Accumulation of genetic alterations characterizes cancer, among which somatic mutations comprise driver mutations and passenger mutations could yield novel cancer‐specific epitopes acknowledged as foreign by autologous T cells, constituting desirable cancer vaccine candidates.^309^ Either type of somatic mutation is capable of altering proteins’ sequence and generating renewed epitopes processed and presented on MHC molecules. Simultaneously, the variant epitopes identified by T cells are referred to as neoepitopes.^310^ Combined with specialized bioinformatics tools, next‐generation sequencing could thoroughly profile the entire spectrum of mutations in cancer and predict renewed epitopes binding to MHC molecules.^311^ Next‐generation sequencing of healthy tissues and tumor biopsies from patients to customize patient‐specific cancer vaccines. Tumor‐specific non‐synonymous single nucleotide variants or short chimeras in protein‐coding genes could be established by comparing tumor and normal DNA sequences. The calculation pipeline is utilized to inspect binding of mutated peptide region to the patient's HLA allele and other features in mutant protein that are thought to be associated with prospective vaccine targets’ prioritization. These data assist in selecting multiple mutations that enable the design of unique new epitope vaccines.^309^, ^312^ Figure 5 summarizes anti‐immunosenescence and anti‐tumor strategies for older people, which together may improve the outlook for older oncology patients.

## 6 CONCLUSION AND PERSPECTIVES

Immunosenescence represents a multifaceted domain characterized by numerous challenges. The intricacies of the immune system, coupled with the multifactorial aspects of aging compounds the complexity of this field. Alterations in various immune cell subsets, including T cells, B cells, and NK cells, diminish the immune system's capacity to respond effectively to novel antigens. Besides, inflammaging is associated with a spectrum of age‐related diseases and also has significant implications for vaccine efficacy and the effectiveness of cancer immunotherapy. Furthermore, individual variations in immunosenescence necessitate personalized treatments tailored to genotype, medical history, and environmental factors. The lack of precise immunosenescence biomarkers also complicates the assessment of anti‐aging therapies. The challenges surrounding immunosenescence therapy cut across multiple domains, including biology, immunology, pathology, and clinical application. Future research must focus on developing effective therapeutic strategies and addressing the aforementioned tasks.

Advances in comprehension of immunosenescence and senescence‐associated diseases have paved the way for potential interventions. Over the past decade, substantial scientific efforts have been directed toward development of pharmacological agents aimed at intervening in senescence process, to ameliorate senescence‐related pathologies. Notably, emerging evidence suggests that the eradicating senescence hallmarks extend healthspan in animal models. This has spurred researchers to devise strategies to either attenuate senescence pathways or eliminate senescent cells directly, employing small molecules or antibodies as senolytic agents. It is becoming utterly evident that health conditions and responsiveness to infections and vaccinations are influenced by circumstantial environmental factors on personal variables such as nutrition, exercise, medication, and co‐morbidities. Tactics to prolong a healthy human lifespan should build on avoiding environmental factors that accelerate immunosenescence, applying lifestyles that promote fitness, and administering relatively non‐specific pleiotropic drugs, or vaccination interventions. More significantly, there are both anti‐immunosenescence and anti‐tumor tactics to improve immune responses in TIME for older cancer patients during treatment. There is also promising work on evaluating combination therapies to maximize efficacy and minimize side effects. While there is a partial consensus on targeting immunosenescence as an innovative paradigm for immunotherapy, this field has not yet produced a coherent perspective on overcoming hurdles and refining patient outcomes in the future by recognizing these clinical complexities and tirelessly striving to surmount inherent constraints.

In recent years, cutting‐edge techniques have been consistently developed, encompassing model systems, bio‐imaging, single‐cell omics, computational analysis, etc. These synthetic methodologies offer significant opportunities to enhance comprehension of complicated senescence processes across multiple scales, ranging from individual molecules to entire organisms. Future challenges and directions also involve standardization of immunosenescence assessment, development of individualized therapeutic strategies as well as systems biology and artificial intelligence in senescence intervention. These promising technologies may shed light on recent advances and future orientations in immunosenescence research, paving the way for more tailored immunosenescent avenues that could yield significant benefits for elderly cancer patients.

## AUTHOR CONTRIBUTIONS

Zaoqu Liu and Xinwei Han provided direction and guidance throughout the preparation of this manuscript. Lulu Zuo and Zhaokai Zhou wrote and edited the manuscript. Zhaokai Zhou reviewed and made significant revisions to the manuscript. Lulu Zuo, Zhaokai Zhou, Shutong Liu, Yuhao Ba, Anning Zuo, Yuqing Ren, Chuhan Zhang, Yukang Chen, Hongxuan Ma, Yudi Xu, Peng Luo, Quan Cheng, Hui Xu, Yuyuan Zhang, and Siyuan Weng collected and prepared the related papers. All authors read and approved the final manuscript.

## CONFLICT OF INTEREST STATEMENT

The authors declare they have no conflicts of interest.

## ETHICS STATEMENT

Not applicable.

## Data Availability

Not applicable.
